# Awareness and preparedness of healthcare workers against the first wave of the COVID-19 pandemic: A cross-sectional survey across 57 countries

**DOI:** 10.1371/journal.pone.0258348

**Published:** 2021-12-22

**Authors:** Nguyen Tien Huy, R. Matthew Chico, Vuong Thanh Huan, Hosam Waleed Shaikhkhalil, Vuong Ngoc Thao Uyen, Ahmad Taysir Atieh Qarawi, Shamael Thabit Mohammed Alhady, Nguyen Lam Vuong, Le Van Truong, Mai Ngoc Luu, Shyam Prakash Dumre, Atsuko Imoto, Peter N. Lee, Dao Ngoc Hien Tam, Sze Jia Ng, Mohammad Rashidul Hashan, Mitsuaki Matsui, Nguyen Tran Minh Duc, Sedighe Karimzadeh, Nut Koonrungsesomboon, Chris Smith, Sharon Cox, Kazuhiko Moji, Kenji Hirayama, Le Khac Linh, Kirellos Said Abbas, Tran Nu Thuy Dung, Tareq Mohammed Ali AL-Ahdal, Emmanuel Oluwadare Balogun, Nguyen The Duy, Mennatullah Mohamed Eltaras, Trang Huynh, Nguyen Thi Linh Hue, Bui Diem Khue, Abdelrahman Gad, Gehad Mohamed Tawfik, Kazumi Kubota, Hoang-Minh Nguyen, Dmytro Pavlenko, Vu Thi Thu Trang, Le Thuong Vu, Tran Hai Yen, Nguyen Thi Yen-Xuan, Luong Thi Trang, Vinh Dong, Akash Sharma, Vu Quoc Dat, Mohammed Soliman, Jeza Abdul Aziz, Jaffer Shah, Pham Dinh Long Hung, Yap Siang Jee, Dang Thuy Ha Phuong, Tran Thuy Huong Quynh, Hoang Thi Nam Giang, Vy Thi Nhat Huynh, Nguyen Anh Thi, Nacir Dhouibi, Truc Phan, Vincent Duru, Nguyen Hai Nam, Sherief Ghozy, contributors of the TMGH-Global COVID-19 Collaborative

**Affiliations:** 1 School of Tropical Medicine and Global Health, Nagasaki University, Nagasaki, Japan; 2 Department of Disease Control, Faculty of Infectious & Tropical Diseases, London School of Hygiene & Tropical Medicine, London, United Kingdom; 3 Faculty of Medicine, Pham Ngoc Thach University, Ho Chi Minh City, Vietnam; 4 Faculty of Medicine, Islamic University of Gaza, Gaza Strip, Palestine; 5 School of Biotechnology, International University—Vietnam National University, Ho Chi Minh City, Vietnam; 6 Lower Westchester Medical Associates, P.C., Mount Vernon, NY, United States of America; 7 Faculty of medicine, University of Gezira, Wad Medani, Sudan; 8 University of Medicine and Pharmacy at Ho Chi Minh City, Ho Chi Minh City, Vietnam; 9 Traditional Medicine Hospital of Ministry of Public Security, Vietnam; 10 Department of Internal Medicine, University of Medicine and Pharmacy at Ho Chi Minh City, Ho Chi Minh, Vietnam; 11 Department of Immunogenetics, Institute of Tropical Medicine, Nagasaki University, Nagasaki, Japan; 12 P.N. Lee Statistics and Computing Ltd., Sutton, United Kingdom; 13 Asia Shine Trading & Service CO. LTD., Ho Chi Minh City, Vietnam; 14 Hospital Enche’ Besar Hajjah Khalsom, Johor, Malaysia; 15 Government of the People’s Republic of Bangladesh—Ministry of Health and Family Welfare, Dhaka, Bangladesh; 16 School of Medicine, Sabzevar University of Medical Sciences, Sabzevar, Iran; 17 Department of Pharmacology, Faculty of Medicine, Chiang Mai University, Chiang Mai, Thailand; 18 London School of Hygiene & Tropical Medicine, London, United Kingdom; 19 Institute of Tropical Medicine, Nagasaki University, Nagasaki, Japan; 20 VinUniversity, College of Health Sciences, Hanoi, Vietnam; 21 Faculty of Medicine, Alexandria University, Alexandria, Egypt; 22 Department of Public Health, Faculty of Medicine, Jordan University of Science and Technology, Ar-Ramtha, Jordan; 23 Department of Biochemistry and African Center of Excellence on Neglected Tropical Diseases and Forensic Biotechnology, Ahmadu Bello University, Zaria, Nigeria; 24 Department of Gyn. Endocrinology and Reproductive Medicine, University Hospital Giessen and Marburg, Marburg, Germany; 25 Philipps University Marburg, Marburg, Germany; 26 Faculty of Medicine for Girls, Al-Azhar University, Cairo, Egypt; 27 Nguyen Trai Hospital, Ho Chi Minh, Vietnam; 28 Ain Shams University, Cairo, Egypt; 29 Faculty of Medicine, Ain Shams University, Cairo, Egypt; 30 Yokohama City University, Yokohama, Japan; 31 Bogomolets National Medical University, Kyiv, Ukraine; 32 National Hospital of Traditional Medicine, Hanoi, Vietnam; 33 Cambridge University Hospital Foundation Trust, Cambridge, United Kingdom; 34 Danang Oncology Hospital, Danang, Vietnam; 35 American University of the Caribbean, Cupe Coy, Saint Maarten, United States of America; 36 University College of Medical Sciences & Guru Teg Bahadur Hospital, Dilshad Garden, Delhi, India; 37 Department of Infectious Diseases, Hanoi Medical University, Hanoi, Vietnam; 38 Faculty of Medicine, Zagazig University, Zagazig, Egypt; 39 Medical Laboratory Science, College of Health Science, University of Human Development, Kurdistan Region, Sulaimani, Iraq; 40 Baxshin Research Training Organization, Baxshin Hospital, Kurdistan Region, Sulaimani, Iraq; 41 Drexel University College of Medicine, Philadelphia, Pennsylvania, United States of America; 42 School of Medical Sciences, Universiti Sains Malaysia, Kelantan, Malaysia; 43 Center for Biomedical Research, Pham Ngoc Thach University of Medicine, Vietnam; 44 Kansai Medical University, Osaka, Japan; 45 Institute of Research and Development, Duy Tan University, Da Nang, Vietnam; 46 Faculty of Medicine and Pharmacy, University of Da Nang, Da Nang, Vietnam; 47 Faculty of Medicine, University of Debrecen, Debrecen, Hungary; 48 Toulouse III Paul Sabatier University, Toulouse, France; 49 Faculty of Medicine of Tunis, University of Tunis El Manar, Tunis, Tunisia; 50 Vinmec International Hospital, Hanoi, Vietnam; 51 Department of Parasitology and Entomology, Nnamdi Azikiwe University, Awka, Nigeria; 52 Division of Hepato-Biliary-Pancreatic Surgery and Transplantation, Department of Surgery, Graduate School of Medicine, Kyoto University, Kyoto, Japan; 53 Faculty of Medicine, Mansoura University, Mansoura, Egypt; University of Haifa, ISRAEL

## Abstract

**Background:**

Since the COVID-19 pandemic began, there have been concerns related to the preparedness of healthcare workers (HCWs). This study aimed to describe the level of awareness and preparedness of hospital HCWs at the time of the first wave.

**Methods:**

This multinational, multicenter, cross-sectional survey was conducted among hospital HCWs from February to May 2020. We used a hierarchical logistic regression multivariate analysis to adjust the influence of variables based on awareness and preparedness. We then used association rule mining to identify relationships between HCW confidence in handling suspected COVID-19 patients and prior COVID-19 case-management training.

**Results:**

We surveyed 24,653 HCWs from 371 hospitals across 57 countries and received 17,302 responses from 70.2% HCWs overall. The median COVID-19 preparedness score was 11.0 (interquartile range [IQR] = 6.0–14.0) and the median awareness score was 29.6 (IQR = 26.6–32.6). HCWs at COVID-19 designated facilities with previous outbreak experience, or HCWs who were trained for dealing with the SARS-CoV-2 outbreak, had significantly higher levels of preparedness and awareness (p<0.001). Association rule mining suggests that nurses and doctors who had a ’great-extent-of-confidence’ in handling suspected COVID-19 patients had participated in COVID-19 training courses. Male participants (mean difference = 0.34; 95% CI = 0.22, 0.46; p<0.001) and nurses (mean difference = 0.67; 95% CI = 0.53, 0.81; p<0.001) had higher preparedness scores compared to women participants and doctors.

**Interpretation:**

There was an unsurprising high level of awareness and preparedness among HCWs who participated in COVID-19 training courses. However, disparity existed along the lines of gender and type of HCW. It is unknown whether the difference in COVID-19 preparedness that we detected early in the pandemic may have translated into disproportionate SARS-CoV-2 burden of disease by gender or HCW type.

## Introduction

Coronavirus disease 2019 (COVID-19) is caused by severe acute respiratory syndrome coronavirus-2 (SARS-CoV-2). The transmission intensity is partially attributable to cycles of community spread [1–4] and healthcare workers (HCWs) are at particular risk. During the initial wave of transmission in China, 3.8% of COVID-19 patients were HCWs, whereas 9.0% of cases were HCWs in Italy [5, 6]. As of 30 November 2021, 770,536 HCWs in the United States had been infected; death status is available for 489,659 (63.55%) of them, of whom 2,890 had died [7]. SARS-CoV-2 infection has contributed to physical, mental, and emotional exhaustion of HCWs, potentially compromising patient care [8]. Heightening these risks has been the emergence of SARS-CoV-2 variants of concern, namely *Alpha* (B.1.1.7; first isolated in the United Kingdom; 175 countries now with the sequence), *Beta* (B.1.351; South Africa; 113 countries with sequence), *Gamma* (P.1; Brazil; 71 countries with sequence), *Delta* (B.1.617.2; India; 147 countries with sequence), and Omicron (B.1.1.529; South Africa and Botswana; 8 countries with sequence) [9]. COVID-19 awareness and preparedness among HCWs are vital to preventing transmission in healthcare facilities (HCFs) and safeguarding the workforce.

Early in the pandemic, the US Centers for Diseases Control and Prevention (CDC), the National Centre for Infectious Diseases in Singapore, and the World Health Organization (WHO) developed COVID-19 preparedness checklists [10]. We adapted these tools to evaluate the awareness and preparedness of HCWs globally with the aim of providing results to decision-makers who may be positioned to retool health systems for subsequent waves of COVID-19 and to inform responses to future infectious disease outbreaks.

## Materials and methods

### Study design and participants

**Fig 1:**
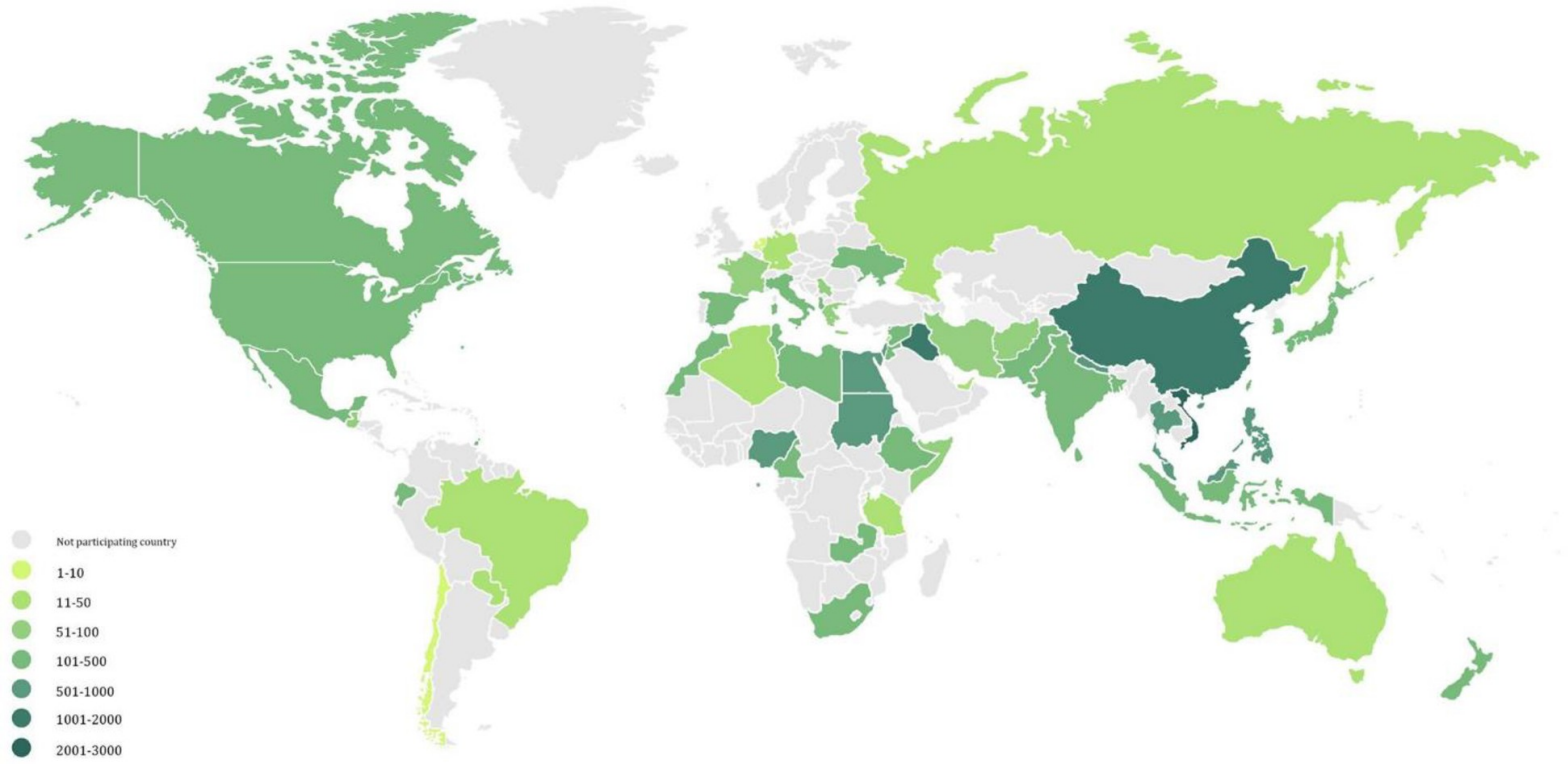
World map showing the distribution of study countries and study participants. Note: Shade of green represents the number of participants per country.

This multicenter, multinational, cross-sectional study of hospital HCWs was conducted between February and May 2020. Surveys were conducted in 371 HCFs across 57 countries and administrative regions (Fig 1 and S1 Table in S1 File). HCWs were invited to participate if they were involved in patient care, handling (or expected to care for) suspected COVID-19 patients, and provided informed written consent that was embedded on the first page of the questionnaire. After reading a descripton of the survey, individuals were asked if they agreed to participate. If they answered “YES” on the electronic form, the survey would begin. Respondents voluntarily participated and could withdraw consent at any time. We used convenience sampling with no restrictions on the number of hospitals and participants per country.

### Questionnaire design and scoring

Our questionnaire contained 32 questions in two sections. The first section consisted of six questions focused on general participant information. The second section included 26 questions related to participant awareness and preparedness against COVID-19. The last question solicited suggestions for improving preparedness. The awareness score was equal to the number of points accumulated over four topics with a maximum of 40 points. The maximum preparedness score was 15 based on responses from 15 questions.

We piloted the initial survey in English among 30 HCWs and revised accordingly. We then translated the instrument into 19 languages: Albanian, Arabic, Bengali, Chinese Mandarin, French, Hindi, Indonesian, Italian, Japanese, Korean, Kurdish, Nepali, Persian, Portuguese, Russian, Spanish, Thai, Urdu, and Vietnamese. We reverse-translated these versions, pre-tested them, and amended the final text as necessary. We used Cronbach’s alpha to estimate the reliability of single-administration test scores. This produced generally acceptable measures of 0.91 for preparedness, 0.61 for awareness, and 0.65 overall [11, 12]. Following survey administration, we extracted data, ran data quality checks, calculated overall awareness and preparedness scores, and stratified results by World Bank country classifications of high, upper-middle, lower-middle, and low income [13, 14].

### Statistical analysis

We summarized participant characteristics using median and interquartile ranges for numeric variables, and tabulated the number of participants and percentages for categorical variables. We reviewed outcome measures as histograms and evaluated associations between participant characteristics and outcome measures using a multi-level linear regression model and random effects models. We assigned participants, hospitals, and countries to levels 1, 2, and 3. We then generated results by mean difference (MD) with 95% confidence intervals (CI) and *P-*values. Where at least one component question was not answered, we performed complete-case and imputed-data analyses in the multi-level model. In our complete-case analysis, missing responses were assigned zero points. For our imputed-data analysis, we estimated missing values with multiple imputation-by-chained-equation methods [15]. We included available data in the imputation model with 20 imputed datasets and 20 cycles per dataset.

**Fig 2:**
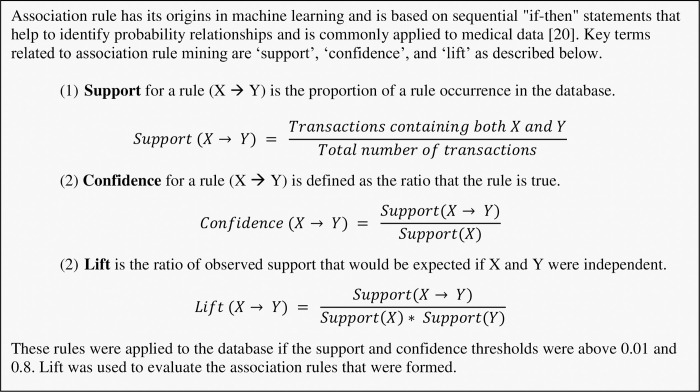
Fundamentals of association rule mining.

We assessed the effect of HCW training courses on awareness and preparedness for managing COVID-19 cases in the hospital by scatter plot and added smoothing lines with Loess method for groups with and without training. We then conducted association rule mining using an algorithm developed *a priori* [16] to identify otherwise undetectable relationships between HCW participation in training courses and their confidence in handling suspected COVID-19 patients, as well as their satisfaction in the medical equipment available in their hospitals for the pandemic response [17]. Participating HCWs by country are illustrated on the world map of Fig 1. We produced using the R ggplot2 package [18] and used R software version 3.6.3 to perform these analyses [19]. Key concepts of association rule mining [20], i.e. support, confidence, and lift, are outlined in Fig 2.

### Ethics approval

The study protocol was approved by the Ethics Committee of the School of Tropical Medicine and Global Health, Nagasaki University, Japan [21], and by all participating healthcare facilities according to local guidelines (S2 Table in S1 File).

## Results

### Socio-demographic characteristics

A total of 17,302 valid surveys out of 24,653 (response rate: 70.18%) were received from HCWs, of which 16,954 reported their gender; 10,843 (64.0%) were women, and 6,045 (35.7%) were men, the remaining 66 participants did not want to report gender. Most participants were younger than 44 years of age (*n* = 14,257, 86.4%) and from Asia (*n* = 11,065, 64.0%). Nurses (*n* = 7,679, 44.7%) and lower-middle income countries (*n* = 7,461, 43.1%) were the most represented (Table 1 and S3 Table in S1 File).

**Table 1:** Sociodemographic characteristics, work experience, and workplace of participating healthcare workers.

Characteristic	Participants, *n* (%)
Sociodemographic	
Median age in years (interquartile range) (*n* = 16,511)	32.0 (27.0, 40.0) ^¶^
Gender (*n* = 16,954)	
Women	10,843 (64.0)
Men	6,045 (35.7)
Prefer not to specify	66 (0.4)
Profession (*n* = 17,195)	
Doctor	6,328 (36.8)
Nurse	7,679 (44.7)
Pharmacist	769 (4.5)
Others	2,419 (14.1)
Work experience and workplace	
Work experience, Range in years (*n* = 14,812)	7.0 (3.0–15.0) ^¶^
Hospital department (*n* = 17,025)	
Emergency department	3,728 (21.9)
Intensive care unit	2,428 (14.3)
Outpatient clinic	2,454 (14.4)
Infectious disease department	1,157 (6.8)
Respiratory department	1,208 (7.1)
Others	7,155 (42.0)
Type of hospital (*n* = 16,335)	
Designated to treat COVID-19	6,847 (41.9)
Not designated but able to treat COVID-19	5,036 (30.8)
Not designated and not able to treat COVID-19	4,452 (27.3)
Previous outbreak experience (*n* = 17,508)	
Experienced any outbreak (*n* = 17,045)	7,508 (44.0)
Experienced SARS outbreak (*n* = 17,045)	3,548 (20.8)
Experienced MERS outbreak (*n* = 17,045)	1,819 (10.7)
Experienced bird flu outbreak (*n* = 17,045)	4,858 (28.5)
Experienced other outbreaks (*n* = 17,045)	1,474 (8.6)
Confirmed SARS-CoV-2 case where you are (*n* = 17,164)	
Yes, in my country	7,071 (41.2)
Yes, in my city	3,095 (18.0)
Yes, in my hospital	2,300 (13.4)
Participated in training course for dealing with COVID-19 (*n* = 17,169)	6,287 (36.6)
How satisfied you are with medical equipment in your hospital (*n* = 17,106)	
Very unsatisfied	1,954 (11.4)
Unsatisfied	3,922 (22.9)
Neutral	4,524 (26.4)
Satisfied	5,049 (29.5)
Very satisfied	1,657 (9.7)
Have confidence in handling suspected COVID-19 patients (*n* = 17,138)	
Not at all	2,849 (16.6)
To a little extent	3,973 (23.2)
To some extent	4,726 (27.6)
To a considerable extent	3,712 (21.7)
To a great extent	1,878 (11.0)

### Workplace characteristics and source of information

Participants had a median of seven years of work experience (*n* = 14,812; interquartile range [IQR] 3.0 to 15.0) and were most commonly from level 3 hospitals (*n* = 11,424; 67.0%) and emergency departments (*n* = 3–728; 21.9%) (Table 1). In total, 44.0% (*n* = 7,508) of HCWs had prior outbreak experience and 13.4% (*n* = 2,300) reported the presence of confirmed cases in their hospitals at the time of survey. Mainstream media was the primary source of information for HCWs (*n* = 13,659; 79.4%), followed by online social networks (*n* = 11,336; 65.9%), and government organizations (*n* = 9,603; 55.9%) (Table 1). Only 36.6% (*n* = 6,287) of HCWs had taken part in a COVID-19 training course. In total, 39.2% of participants were satisfied (29.5%) or very satisfied (9.7%) with available medical equipment. Most HCWs (*n* = 10,316; 84.4%) had some degree of confidence in handling suspected cases.

### COVID-19 preparedness and awareness scores

In total, 15,689 (90.1%) and 16,419 (94.9%) participants completed all questions in the preparedness and awareness sections. There was high agreement in results generated by complete-case versus imputed-data analyses. Specifically, there were significant associations in 21 analyses of complete-case data, and 22 significant associations from imputed-data analyses. Collectively, 19 significant associations were concordant between the two analyses (Table 2).

**Table 2:** Multilevel models for preparedness and awareness scores of participating healthcare workers.

Variable	Complete-case analysis			Imputed data analysis		
	MD	(95% CI)	*P-*value	MD	(95% CI)	*P-*value
Preparedness						
Region						
East Asia & Pacific	*Reference*			*Reference*		
Europe & Central Asia	-1.25	(-2.74, 0.24)	0.099	-1.13	(-2.59, 0.33)	0.128
Latin America & Caribbean	-2.96	(-4.75, -1.17)	**0.001** *	-2.96	(-4.73, -1.20)	**0.001** *
Middle East & North Africa	-3.20	(-4.58, -1.81)	**<0.001** *	-3.27	(-4.64, -1.91)	<0.001*
North America	-2.24	(-5.51, 1.03)	0.180	-3.15	(-5.61, -0.69)	**0.012** *
South Asia	-2.36	(-4.11, -0.61)	**0.008** *	-2.54	(-4.26, -0.82)	**0.004** *
Sub-Saharan Africa	-4.32	(-6.01, -2.62)	**<0.001** *	-4.54	(-6.21, -2.87)	<0.001*
Income level						
High income	*Reference*			*Reference*		
Upper middle income	-0.18	(-1.53, 1.17)	0.794	-0.11	(-1.44, 1.22)	0.874
Lower middle income	-0.87	(-2.41, 0.66)	0.264	-0.78	(-2.29, 0.73)	0.310
Low income	-1.08	(-3.14, 0.97)	0.301	-1.05	(-3.08, 0.97)	0.308
Level of hospital						
1st level	*Reference*			*Reference*		
2nd level	-0.29	(-0.97, 0.39)	0.406	-0.12	(-0.75, 0.52)	0.721
3rd level	0.08	(-0.58, 0.74)	0.805	0.29	(-0.30, 0.87)	0.339
Type of hospital						
Designated to treat COVID-19	*Reference*			*Reference*		
Not designated but able to treat COVID-19	-0.00	(-0.52, 0.51)	0.993	-0.14	(-0.57, 0.29)	0.530
Not designated & unable to treat COVID-19	-0.72	(-1.33, -0.11)	**0.020** *	-0.37	(-0.81, 0.07)	0.098
Age (every 10-year increase)	0.40	(0.28, 0.53)	**<0.001** *	0.41	(0.30, 0.52)	<0.001*
Gender						
Women	*Reference*			*Reference*		
Men	0.35	(0.23, 0.47)	**<0.001** *	0.28	(0.17, 0.38)	<0.001*
Other	-0.64	(-1.71, 0.43)	0.239	-0.60	(-1.34, 0.14)	0.113
Profession						
Doctor	*Reference*			*Reference*		
Nurse	0.66	(0.54, 0.81)	**<0.001** *	0.67	(0.55, 0.79)	<0.001*
Pharmacist	-0.86	(-1.13, -0.59)	**<0.001** *	-0.77	(-1.02, -0.53)	<0.001*
Others	-0.57	(-0.75, -0.39)	**<0.001** *	-0.62	(-0.77, -0.47)	<0.001*
Experience (every 10-year increase)	0.10	(-0.03, 0.24)	0.136	0.06	(-0.06, 0.18)	**0.337**
Experienced any outbreak						
Yes	*Reference*			*Reference*		
No	-0.56	(-0.67, -0.44)	**<0.001** *	-0.52	(-0.62, -0.42)	<0.001*
Confirmed COVID-19 case where you are						
No	*Reference*			*Reference*		
Yes, in my country	0.34	(0.15, 0.53)	**<0.001** *	0.27	(0.10, 0.44)	0.002*
Yes, in my city	0.28	(0.04, 0.53)	**0.025** *	0.20	(-0.01, 0.41)	0.063
Yes, in my hospital	0.65	(0.35, 0.95)	**<0.001** *	0.50	(0.24, 0.75)	<0.001*
Awareness						
Region						
East Asia & Pacific	*Reference*			*Reference*		
Europe & Central Asia	0.81	(-0.79, 2.41)	0.321	0.57	(-0.90, 2.03)	0.448
Latin America & Caribbean	0.08	(-1.82, 1.98)	0.933	0.19	(-1.55, 1.94)	0.830
Middle East & North Africa	-1.37	(-2.86, 0.12)	0.071	-0.91	(-2.30, 0.48)	0.201
North America	0.67	(-2.84, 4.19)	0.707	0.56	(-1.92, 3.04)	0.659
South Asia	-1.31	(-3.19, 0.57)	0.173	-0.97	(-2.73, 0.79)	0.279
Sub-Saharan Africa	-0.56	(-2.38, 1.27)	0.551	-0.18	(-1.89, 1.53)	0.837
Income level						
High income	*Reference*			*Reference*		
Upper middle income	-1.71	(-3.15, -0.26)	**0.020** *	-2.02	(-3.34, -0.70)	0.003*
Lower middle income	-1.48	(-3.19, 0.16)	0.078	-1.54	(-3.06, -0.02)	0.047*
Low income	-1.21	(-3.49, 1.00)	0.283	-1.73	(-3.77, 0.31)	0.097
Level of hospital						
1st level	*Reference*			*Reference*		
2nd level	-0.29	(-1.01, 0.43)	0.425	0.02	(-0.56, 0.60)	0.946
3rd level	0.41	(-0.28, 1.11)	0.242	0.46	(-0.09, 1.01)	0.098
Type of hospital						
Designated to treat COVID-19	*Reference*			*Reference*		
Not designated but able to treat COVID-19	0.17	(-0.37, 0.70)	0.542	0.03	(-0.36, 0.42)	0.892
Not designated & unable to treat COVID-19	-0.32	(-0.96, 0.31)	0.319	-0.15	(-0.59, 0.28)	0.486
Age (every 10-year increase)	-0.16	(-0.34, 0.01)	0.068	-0.02	(-0.16, 0.12)	**0.772**
Gender						
Women	*Reference*			*Reference*		
Men	-0.02	(-0.19, 0.15)	0.791	-0.11	(-0.24, 0.02)	0.103
Other	-0.68	(-2.19, 0.83)	0.375	-0.96	(-1.87, -0.04)	0.040*
Profession						
Doctor	*Reference*			*Reference*		
Nurse	-1.97	(-2.16, -1.78)	**<0.001** *	-1.76	(-1.91, -1.61)	<0.001*
Pharmacist	-1.56	(-1.94, -1.18)	**<0.001** *	-1.26	(-1.57, -0.95)	<0.001*
Others	-2.44	(-2.69, -2.19)	**<0.001** *	-2.08	(-2.28, -1.89)	<0.001*
Experience (every 10-year increase)	0.06	(-0.13, 0.25)	0.547	0.01	(-0.15, 0.16)	**0.953**
Experienced any outbreak						
Yes	*Reference*			*Reference*		
No	-0.49	(-0.66, -0.33)	**<0.001** *	-0.26	(-0.39, -0.13)	<0.001*
Confirmed COVID-19 case where you are						
No	*Reference*			*Reference*		
Yes, in my country	1.53	(1.26, 1.80)	**<0.001** *	1.30	(1.09, 1.52)	<0.001*
Yes, in my city	1.75	(1.40, 2.09)	**<0.001** *	1.57	(1.30, 1.83)	<0.001*
Yes, in my hospital	1.79	(1.38, 2.21)	<0.001*	1.62	(1.30, 1.94)	<0.001*

MD: mean difference; CI: confidence interval

*Statistically significant

#### Preparedness scores

The median preparedness score of all participants was 11.0 (*n* = 17,302; IQR 6.0 to 14.0). Results from the multi-level linear model suggest that socio-demographic characteristics had a significant effect on participant preparedness scores (Table 2). Relative to East Asia and the Pacific, preparedness scores were significantly lower in the complete-case analysis among participants from sub-Saharan Africa (MD -4.32; CI -6.01 to -2.62; *P* < 0.001), the Middle East and North Africa (MD -3.20; CI = -4.58 to -1.81; *P* < 0.001), Latin America and the Caribbean (MD -2.96; CI -4.75 to -1.17; *P* = 0.001), and South Asia (MD -2.36; CI -4.11 to -0.61; *P* = 0.008). Imputed-data from North America also had significantly lower preparedness scores (MD -3.15; CI = -5.61 to -0.69; *P* = 0.012) than those in East Asia and the Pacific region.

There was a significant increase in the participant preparedness score for every 10-year increase in age, whether in completed-case (MD 0.40; CI = 0.28 to 0.53; *P* < 0.001) or imputed-data sets (MD 0.41; CI 0.30 to 0.52; *P* < 0.001). Male participants (MD 0.35; CI 0.23 to 0.47; *P* < 0.001) and nurses (MD 0.66; CI 0.54 to 0.81; *P* < 0.001) had higher preparedness scores compared to women and doctors (Table 2). The type of HCF and prior pandemic experience had a significant effect on preparedness scores; HCWs at hospitals who were not designated and not able to treat COVID-19 patients had significantly lower preparedness scores (MD -0.72; CI -1.33 to -0.11; *P* = 0.020) in the complete-case analysis, a finding that did not persist with data imputations (MD -0.37; CI -0.81 to 0.07; *P* = 0.098). Participants with no previous outbreak experience had significantly lower preparedness scores in both completed-case (MD -0.56; CI -0.67 to -0.44; *P* < 0.001) and imputed-data (MD -0.52; CI -0.62 to -0.42; *P* < 0.001) analyses. Participants from hospitals with confirmed COVID-19 case(s) had the highest preparedness score in the complete-case analysis compared to hospitals without confirmed case(s) (MD 0.65; CI 0.35 to 0.95; *P* < 0.001), a finding that was also reflected in the imputed-data analysis (Table 2). The preparedness score for participants who had COVID-19 training averaged 12.90 ±2.97 compared to participants without training 7.98 ±4.33 (*P* = 0.001).

#### Awareness scores

The median awareness score was 29.6 of 40 possible points (*n* = 17,302; IQR 26.6 to 32.6). Table 2 shows results from the multilevel linear model. Among socio-demographic characteristics, only income level and profession had a significant effect on awareness scores. Participants from upper-middle income countries (MD -1.71; CI -3.15 to -0.26; *P* = 0.020) had significantly lower awareness scores compared to those from high income countries by complete-case analysis, a finding consistent with imputed data. Doctors served as the reference group, having the highest awareness scores, followed by pharmacists (MD -1.56; CI -1.94 to -1.18; *P* < 0.001), nurses (MD -1.97; CI -2.16 to -1.78; *P* < 0.001), and other professions (MD -2.44; CI -2.69 to -2.19; *P* < 0.001); results were also significant in the imputed-data analysis (Table 2). Individuals with no previous outbreak experience had significantly lower awareness scores than those with experience (MD -0.49; CI -0.66 to -0.33; *P* < 0.001) in the complete-case analysis, which was also reflected in the imputed data. Similarly, complete-case analyses showed participants with confirmed COVID-19 case(s) in their hospital, city, or country had significantly higher awareness scores (MD 1.79; CI 1.38 to 2.21; *P* < 0.001; 1.75; CI 1.40 to 2.09; *P* < 0.001; and 1.53; CI 1.26 to 1.80; *P* < 0.001), findings that were similar in the imputed-data (Table 2). HCWs who received COVID-19 training had a total awareness score of 29.3 ±4.00 which was significantly higher than a score of 28.9 ± 5.48 among HCWs without training (MD 0.40; CI: 0.25 to 0.56, *P* < 0.001).

**Fig 3:**
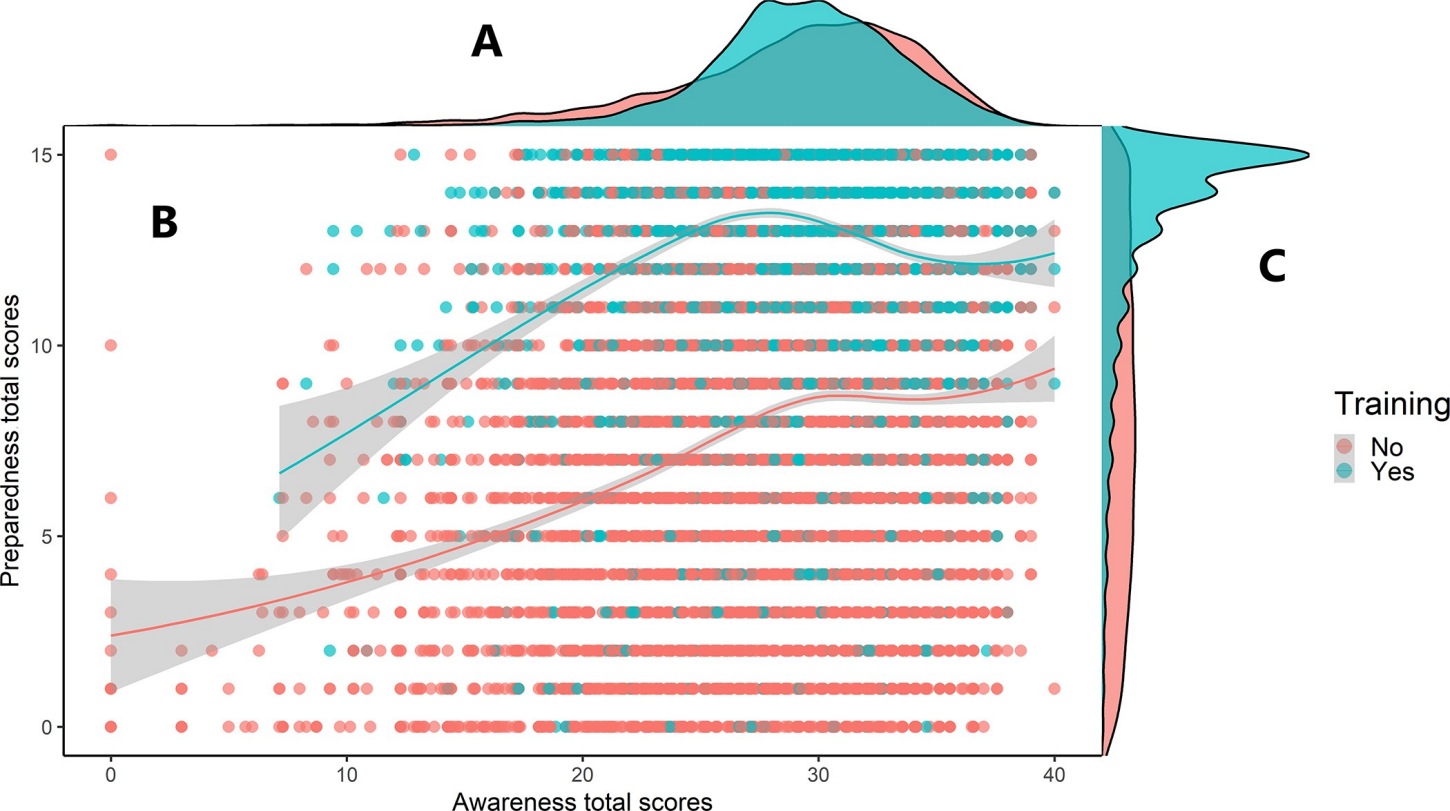
Two multivariate cowplots illustrating the effect of training on preparedness and awareness. Part A illustrates the distribution of awareness scores. Part B shows individual total scores of preparedness and awareness. The center lines were computed using Loess method with the shadow representing their 95% confidence intervals. Part C illustrates the distribution of preparedness scores.

Fig 3 illustrates positive correlations between awareness and preparedness scores for those who received COVID-19 training and those who did not. A high number of participants with awareness scores between 28 and 30 were in the trained group, with preparedness scores between 13 and 15 points. No similar concentration was observed in the non-trained group.

**Fig 4:**
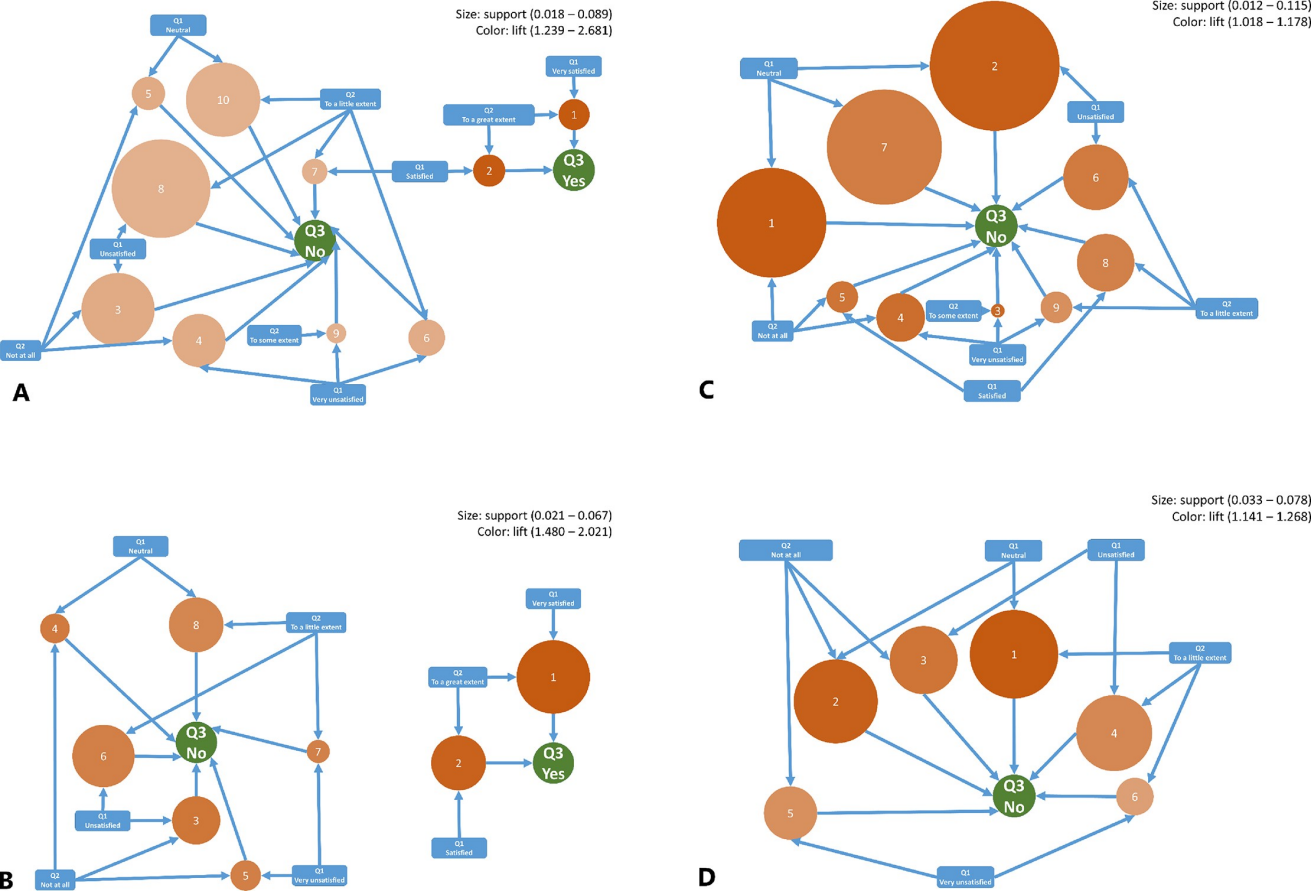
A-D. Association rules between HCWs training, confidence in handling suspected COVID-19 patients, and satisfaction in the current medical equipment for the management of COVID-19 in HCWs hospitals. Note: Numbered circles represent the generated rules and their properties, where the size represents the rate at which these rules occur in the data (support), and the color represents the lift value in which the darker the circle, the higher the probability that the antecedents and consequents are connected. The arrows pointing toward the circle are the rule inputs (antecedents), and the arrows coming out are the rule outputs (consequents). Fig 4A–4D show the findings for doctors, nurses, pharmacists, and other HCWs, respectively, and visually display the generated rules shown in S4 Table in S1 File, which also contains information about the confidence index of these rules. For detailed interpretation of the figures, refer to S1 Text in S1 File.

The mining algorithm produced 33 rules (Fig 4). Summaries of the support, confidence, and lift for each association rule are presented in S4 Table in S1 File. Nurses and doctors who were confident in handling suspected COVID-19 patients (to a ’great extent’) and satisfied in the current medical equipment for COVID-19 management (’very satisfied’) had participated in any training courses for dealing with COVID-19 with support levels of 0.067 and 0.028, confidence levels of 0.925 and 0.873, and lift levels of 2.021 and 2.681. Less confidence (’little extent’ or ’not confident’) and less satisfaction (’unsatisfied’ or ’very unsatisfied’) implied that they had not participated in any COVID-19 training with support levels of 0.021 and 0.089, confidence between 0.830 and 0.932, and lift of 1.244 and 1.685. Fig 4A illustrates relationships identified with association rule mining among survey responses from participant doctors. Question 1 queried the satisfaction level of doctors in the current medical equipment for the management of COVID-19; Question 2 elicited confidence of doctors in handling suspected COVID-19 patients; Question 3 accounted for participation of doctors in any training courses that dealt with COVID-19. Fig 4B–4D illustrate association rule mining among nurses, pharmacists, and other HCWs.

Q1: Satisfaction level in the current medical equipment for the management of COVID-19.

Q2: Confidence level in handling suspected COVID-19 patients.

Q3: Participation in any training courses for dealing with COVID-19 outbreak.

### Interpretation

The high level of agreement between complete-case data analysis and imputed-data analysis suggests that missing data did not skew our results. Overall, we found HCWs to be prepared for and aware of the COVID-19 pandemic to some extent. This level was the highest among nurses (for preparedness) and doctors (for awareness), findings that differ from earlier reports, particularly in regards to preparedness levels [22, 23]. Regardless, we might expect nurses to be more prepared since they were encourage to take on expanded roles in 2018 by international and national agencies (e.g., World Health Organization, International Council of Nurses, American Nurses Association, CDC) in rapid mobilization of responses in any pandemic [24]. We found an association between older age and greater preparedness in contrast to other studies outside infectious disease management [25–27].

Although disease burden continues to spread regardless of economic status, national wealth was significantly associated with the level of preparedness, which is consistent with reports from the literature. Studies from Saudi Arabia, for instance, showed higher preparedness levels from Yemen and Palestinian Territories [28–30]. This is reflected in the capacity of high-income countries to deploy large-scale testing in a short time period. An estimated 56.0% of HCWs lacked outbreak experience and, not surprisingly, their preparedness and awareness scores were significantly lower than measures among HCWs with some experience of SARS, MERS, and avian influenza outbreaks. Higher scores among older HCWs underscores the potential for sharing experiences between staff and hospitals, and the importance of preserving institutional memory. This experience can be tapped to foster South-South cooperation and form the basis of South-North exchange. Prior experience with SARS enabled countries and territories (e.g. Vietnam, Taiwan, and Hong Kong) to combat COVID-19 successfully in early stages [31–33].

Vietnam, despite being a low-resource country, and Hong Kong, with its relative proximity to Wuhan (923 km or 573 miles) and large numbers of international travelers, were both able to control the first wave of the pandemic by deploying a comprehensive government response that included travel bans and aggressive quarantine strategies, suspension of non-essential business, transportation, and schooling, and prioritizing rapid improvements in health care facilities [33, 34]. In Vietnam, following the 2003 outbreak of SARS and H1N1 in 2009, the Vietnamese Centers for Disease Control and Prevention led a national effort to upgrade infectious disease facilities and related equipment. Taiwan applied lessons from the SARS outbreak and had in place a framework for an integrated response to future pandemics [35].

General knowledge of COVID-19 is crucial for HCWs to be equipped adequately and manage suspected or confirmed cases. However, it is concerning that the majority of participants relied on the mainstream media (79.4%) and social media (65.9%) as their primary source of information. The WHO has warned that an ‘infodemic’ of widespread misinformation is a serious concern [36]. HCWs must carefully evaluate information to ensure that it is grounded in evidence. While the HCW interpersonal sources used may not be decisive, group sensemaking is especially important and time-sensitive when the international health community is suffering. However, HCW should always evaluate the credibility of the information by double-checking with trusted sources like WHO and other regional and national health agencies. The emergence of coronavirus variants underscores this importance. Government authorities need to provide accurate and timely guidance to HCWs. At the time of the survey, up to 30% of respondents could not identify some known COVID-19 symptoms or preventive measures to minimize the transmission (S5 Table in S1 File).

In Vietnam, many measures were used to disseminate accurate and updated information. The Vietnamese Ministry of Health, for example, regularly updated its website with news and the latest control measures. They also deployed mobile apps that provided official daily notices of detected clusters; the local app Zalo had approximately 100 million users at one time, contributing to awareness raising and preparedness of Vietnamese HCWs with daily and sometimes hourly updated news about COVID-19 [33].

In our survey, female HCWs had lower preparedness/awareness scores than male counterparts, which is consistent with reports from another preparedness study [29]. Female HCWs have had vastly higher rates of infection compared to males in Spain (*n* = 21,392; 75.5%), Italy (*n* = 14,350; 69.0%), and the United States (*n* = 6,776; 73.0%) [37, 38]. It is difficult to know whether the difference in preparedness between male and female HCWs that we detected in our global survey early in the pandemic later translated to female HCWs being disproportionately infected by SARS-CoV-2. Regardless, female HCWs need to be afforded equal training opportunities. Although specific training was associated with greater preparedness scores, this was accompanied by a minimal increase in awareness scores, suggesting that further improvements in training may be required. Similar to other studies, we observed an association between training and confidence levels of HCWs [39–41]. Thus, the introduction of training courses should be an essential part of preparedness and response plans.

#### Limitations

Firstly, this study was mainly conducted online among HCWs at a relatively early stage of the pandemic. This does not inherently bias our results, but it does limit the generalizability to facilities and HCWs that have Internet access. In addition, the high participation rates among HCWs in Asia, a region with prior experience managing the SARS and MERS epidemics, could mean our results overstate the true COVID-19 awareness and preparedness of HCWs in other regions of the world at the time of the survey. Finally, there is always a potential risk of bias in data collection when surveys are not completed in their entirety. We overcame this, however, by performing complete-case and imputed-data analyses in our multi-level models.

In addition, at the beginning of our study, we developed questions to survey HCWs preparedness mainly from the CDC’s healthcare professional SARS-CoV-2 preparedness checklist, which was considered the most up to date at the time [10]. The checklist had the same structure and content as the HCW preparedness checklist for MERS-CoV, which has not been updated since July 2013 [42]. Given the fact that our survey was based on the CDC’s latest guidance and our intention was to reach as many HCWs as possible around the globe, our survey questionnaire was translated into 19 different languages and distributed to 17,302 HCWs in 57 countries over a short period of time. However, as the COVID-19 became a pandemic, the CDC updated the checklist to be more specific for the disease. The initial checklist is now only applied for the transportation and admission of patients with suspected or confirmed COVID-19 [10]. Regarding the questions themselves to assess the HCWs’ awareness, we used multiple choice format to survey their awareness on symptoms of COVID-19. At the time of our survey, “red eyes” was not recognized as a manifestation of COVID-19 and, as a result, a majority of HCWs chose “false” for this symptom which was considered the right answer to the question. However, the answer should be “true” now due to more recent understandings of the disease [43].

Our study provides a first glance of HCWs worldwide and their preparedness toward a new emergent prone-disease pandemic. Follow-up research is suggested to compare the differences between current knowledge of HCWs and to measure the differences, and possible improvements, in awareness and preparedness of HCPs over time as the COVID-19 pandemic has gone through three waves.

## Conclusion

We found an acceptable level of awareness and preparedness among HCWs specific to COVID-19 during the time of the survey, although there was disparity along gender lines, type of HCW, and previous experience of similar outbreaks. Training opportunities need to be gender-equitable to safeguard the workforce and stem SARS-CoV-2 transmission in HCF. Preparedness may be facilitated by increased South-South and South-to-North knowledge exchange to benefit from similar experiences of previous disease outbreaks.

## Supporting information

S1 File (DOCX)
