# Supplementary material for: Awareness and preparedness of healthcare workers against the first wave of the COVID-19 pandemic: A cross-sectional survey across 57 countries
Source: PLoS One. 2021 Dec 22;16(12):e0258348. doi: 10.1371/journal.pone.0258348 (PMC8694437; doi:10.1371/journal.pone.0258348)
Supplement: S1 File — (DOCX) [file pone.0258348.s001.docx]

**S1 File**

**Awareness and preparedness of healthcare workers against the first wave of the COVID-19 pandemic: a cross-sectional survey across 57 countries**

Nguyen Tien Huy, R Matthew Chico, Vuong Thanh Huan, Hosam Waleed Shaikhkhalil, Vuong Ngoc Thao Uyen, Ahmad Taysir Atieh Qarawi, Shamael Thabit Mohammed Alhady, Nguyen Lam Vuong, Le Van Truong, Mai Ngoc Luu, Shyam Prakash Dumre, Atsuko Imoto, Peter N Lee, Dao Ngoc Hien Tam, Sze Jia Ng, Mohammad Rashidul Hashan, Mitsuaki Matsui, Nguyen Tran Minh Duc, Sedighe Karimzadeh, Nut Koonrungsesomboon, Chris Smith, Sharon Cox, Kazuhiko Moji, Kenji Hirayama^19^, Le Khac Linh^20^, Kirellos Said Abbas, Tran Nu Thuy Dung, Tareq Mohammed Ali AL-Ahdal, Emmanuel Oluwadare Balogun, Nguyen The Duy, Mennatullah Mohamed Eltaras, Trang Huynh, Nguyen Thi Linh Hue, Bui Diem Khue, Abdelrahman Gad, Gehad Mohamed Tawfik, Kazumi Kubota, Hoang-Minh Nguyen, Dmytro Pavlenko, Vu Thi Thu Trang, Le Thuong Vu, Tran Hai Yen, Nguyen Thi Yen-Xuan, Luong Thi Trang, Vinh Dong, Akash Sharma, Vu Quoc Dat, Mohammed Soliman, Jeza Abdul Aziz, Jaffer Shah, Pham Dinh Long Hung, Yap Siang Jee, Dang Thuy Ha Phuong, Tran Thuy Huong Quynh, Hoang Thi Nam Giang, Vy Thi Nhat Huynh, Nguyen Anh Thi, Nacir Dhouibi, Truc Phan, Vincent Duru, Nguyen Hai Nam, Sherief Ghozy, *and contributors of the TMGH-Global COVID-19 Collaborative*.

[**Table S1:**](#Table_S1) Global distribution of hospitals and healthcare workers included in the study

[**Table S2:**](#Table_S2) List of hospitals that provided the Institutional Review Board approvals

[**Table S3:**](#Table_S3) Sociodemographic characteristics, work experience, workplace, and source of information and related attitudes of participating healthcare workers

[**Table S4:**](#Table_S4) Summaries of support, confidence, lift values for the generated association rules

[**Table S5:**](#Table_S5) Questions used to assess preparedness of healthcare workers and response frequency

[**Text S1**](#Text_S1)**:** Detailed interpretation of Fig 4

[**Text S2:**](#Text_S2) English version of questionnaire used in cross-sectional survey

**[Text S3:](#Text_S3)** Contributors of TMGH-Global COVID-19 Collaborative

| **Table S1: Global distribution of hospitals and healthcare workers included in the study** | | | |
| --- | --- | --- | --- |
| **Country/Territory**  **Hospital ID** | **Hospital name** | **No. HCWs** | **% of total** |
| Afghanistan (1) | | | |
| 1 | Aliabad Teaching Hospital | 9 | 0.05 |
| 2 | Blossom Healthcare Center | 17 | 0.10 |
| 3 | Jamhuriat Hospital | 15 | 0.09 |
| 4 | Maiwand Teaching Hospital | 17 | 0.10 |
| 5 | Rabia Balkhi Hospital | 12 | 0.07 |
| 6 | Wazir Mohammad Akbar Khan | 12 | 0.07 |
| Albania (2) | | | |
| 7 | Gynecological Obstetric University Hospital “Koço Gliozheni" | 122 | 0.71 |
| Algeria (3) | | | |
| 8 | Frantz Fanon Hospital Blida | 14 | 0.08 |
| Australia (4) | | | |
| 9 | Westmead Hospital | 34 | 0.20 |
| Bangladesh (5) | | | |
| 10 | Chittagong Medical College Hospital | 50 | 0.29 |
| 11 | Mymensingh Medical College Hospital | 31 | 0.18 |
| 12 | Rajshahi Medical College Hospital | 21 | 0.12 |
| 13 | Sylhet M.A.G Osmani Medical College Hospital | 24 | 0.14 |
| Brazil (6) | | | |
| 14 | Regional Hospitals: Walter Alberto Pecóits & Sudoeste do Paraná | 23 | 0.13 |
| Cameroon (7) | | | |
| 15 | Banyo District Hospital | 33 | 0.19 |
| 16 | Centre Medicale d'Arrondissement de Ngaoundal | 11 | 0.06 |
| 17 | Regional Hospital of Buea | 60 | 0.35 |
| Canada (8) | | | |
| 18 | Kingston General Hospital | 41 | 0.24 |
| 19 | Hôtel-Dieu de Montréal Hospital | 92 | 0.53 |
| 20 | Providence Care Hospital | 91 | 0.53 |
| 21 | Queen's Family Health Team | 11 | 0.06 |
| Chile (9) | | | |
| 22 | Hospital Hernan Henriquez Aravena | 8 | 0.05 |
| China (10) | | | |
| 23 | Qilu Hospital of Shandong University | 507 | 2.93 |
| 24 | Shandong Provincial Qianfoshan Hospital | 276 | 1.60 |
| 25 | Shandong Provincial Third Hospital | 147 | 0.85 |
| 26 | Subei People's Hospital | 62 | 0.36 |
| 27 | The People's Hospital of Zouping City | 146 | 0.84 |
| 28 | The People's Hospital of Rizhao | 260 | 1.50 |
| 29 | The Second Hospital of Shandong University | 100 | 0.58 |
| 30 | Yan an Hospital Affiliated to Kunming Medical University | 12 | 0.07 |

| **Table S1: Global distribution of hospitals and healthcare workers included in the study (continued)** | | | |
| --- | --- | --- | --- |
| **Country/Territory**  **Hospital ID** | **Hospital name** | **No. HCWs** | **% of total** |
| 31 | Yangzhou First People's Hospital | 62 | 0.36 |
| 32 | Yangzhou Hospital of Traditional Chinese Medicine | 67 | 0.39 |
| 33 | Yidu Central Hospital of Weifang | 221 | 1.28 |
| **Ecuador** (11) | | | |
| 34 | Clínica Infes | 14 | 0.08 |
| 35 | Hospital de los Valles | 47 | 0.27 |
| 36 | Hospital General Docente de Calderón | 89 | 0.51 |
| 37 | Hospital Vozandes | 40 | 0.23 |
| **Egypt** (12) | | | |
| 38 | Abassia Fever Hospital | 16 | 0.09 |
| 39 | Ain Shams University Hospital (El-Demerdash) | 96 | 0.55 |
| 40 | Ain Shams University Specialized Hospital | 35 | 0.20 |
| 41 | Al Hayat Hospital | 12 | 0.07 |
| 42 | Al-Azhar University Hospital | 45 | 0.26 |
| 43 | Al-Naql El-Bari Hospital | 11 | 0.06 |
| 44 | Al-Sadr Hospital of Fayoum (Chest Hospital in Fayoum) | 23 | 0.13 |
| 45 | Al-Safwa Specialist Hospital | 18 | 0.10 |
| 46 | Al-Zahraa Hospital | 15 | 0.09 |
| 47 | Alexandria Main Hospital (Almiri) | 16 | 0.09 |
| 48 | Alexandria Scientific Research Hospital | 12 | 0.07 |
| 49 | Assiut Main University Hospital | 38 | 0.22 |
| 50 | Assiut Pediatrics University Hospital | 15 | 0.09 |
| 51 | Aswan University Hospital | 94 | 0.54 |
| 52 | Atsa Central Hospital | 16 | 0.09 |
| 53 | Burg El-Arab University Hospital | 11 | 0.06 |
| 54 | Damietta Fever Hospital | 49 | 0.28 |
| 55 | El Shatby Hospital for Pediatrics and Newborn | 28 | 0.16 |
| 56 | El-Mowasah University Hospital | 8 | 0.05 |
| 57 | El-Talaba University Hospital | 12 | 0.07 |
| 58 | Gerdo Primary Health Centre | 12 | 0.07 |
| 59 | Internal Medicine Hospital of Fayoum University | 29 | 0.17 |
| 60 | Manshe'at El-Bakry Hospital | 21 | 0.12 |
| 61 | Mostafa Hassan Teaching Hospital | 16 | 0.09 |
| 62 | New Assuit University Hospital | 10 | 0.06 |
| 63 | Om El-Qosor University Hospital | 15 | 0.09 |
| 64 | Omooma Hospital | 11 | 0.06 |
| 65 | Smouha University Hospital | 21 | 0.12 |
| 66 | Tanta University Educational Hospital | 43 | 0.25 |
| 67 | Zagazig University Hospital | 18 | 0.10 |

| **Table S1: Global distribution of hospitals and healthcare workers included in the study (continued)** | | | |
| --- | --- | --- | --- |
| **Country/Territory**  **Hospital ID** | **Hospital name** | **No. HCWs** | **% of total** |

| **Ethiopia** (13) | | | |
| --- | --- | --- | --- |
| 68 | Dessie Referral Hospital | 23 | 0.13 |
| 69 | Dil-Chora Hospital | 20 | 0.12 |
| 70 | Jimma Medical Center | 18 | 0.10 |
| 71 | Selam General Hospital | 10 | 0.06 |
| 72 | Tikur Anbessa Specialized Hospital | 54 | 0.31 |
| 73 | Yekatit-12 Hospital | 36 | 0.21 |
| 74 | Zewditu Memorial Hospital | 45 | 0.26 |
| **France** (14) | | | |
| 75 | Hospital Group Pellegrin Chu De Bordeaux | 47 | 0.27 |
| 76 | Pitié-Salpêtrière Hospital | 28 | 0.16 |
| **Germany** (15) | | | |
| 77 | University Hospital of Giessen | 18 | 0.10 |
| **Greece** (16) | | | |
| 78 | Athens Naval and Veterans Hospital | 14 | 0.08 |
| **Guatemala** (17) | | | |
| 79 | Liga Nacional contra el Cáncer INCAN | 88 | 0.51 |
| **Hong Kong** (18) | | | |
| 80 | Pamela Youde Nethersole Eastern Hospital | 43 | 0.25 |
| 81 | Queen Mary Hospital | 75 | 0.43 |
| **India** (19) | | | |
| 82 | Bangalore Medical College and Research Institute | 152 | 0.88 |
| 83 | Computerized Pathology Lab | 17 | 0.10 |
| 84 | Indira Gandhi Institute of Dental Sciences, Kerala, India | 44 | 0.25 |
| 85 | RDT Hospital, Bathalapalli, AP, India | 47 | 0.27 |
| 86 | Smt Kashibai Navale Medical College and General Hospital, Pune | 25 | 0.14 |
| **Indonesia** (20) | | | |
| 87 | Bangli Hospital-Bali | 25 | 0.14 |
| 88 | Bhayangkara Lemdiklat Polri-Jakarta | 26 | 0.15 |
| 89 | Panti Rapih Hospital, Yogyakarta | 19 | 0.11 |
| 90 | Sungai Dareh Hospital-West Sumatra | 25 | 0.14 |
| 91 | Yuliddin Away Hospital-Aceh | 17 | 0.10 |
| **Iran** (21) | | | |
| 92 | Amir Al Momenin Hospital | 20 | 0.12 |
| 93 | Ayatollah Khansari Hospital | 28 | 0.16 |
| 94 | Valiasr Hospital | 22 | 0.13 |
| **Iraq** (22) | | | |
| 95 | Al Hussain Teaching Hospital | 39 | 0.23 |
| 96 | AL Rometha Hospital | 30 | 0.17 |
|  |  |  |  |

| **Table S1: Global distribution of hospitals and healthcare workers included in the study (continued)** | | | |
| --- | --- | --- | --- |
| **Country/Territory**  **Hospital ID** | **Hospital name** | **No. HCWs** | **% of total** |

| 97 | Al Wasiti Educational Hospital | 50 | 0.29 |
| --- | --- | --- | --- |
| 98 | Al-Hilla Teaching Hospital | 30 | 0.17 |
| 99 | Al-Hindiya General Hospital | 30 | 0.17 |
| 100 | Al-Kadhumain Teaching Hospital | 37 | 0.21 |
| 101 | Al-Khudhr General Hospital | 33 | 0.19 |
| 102 | Al-Yarmuk Teaching Hospital | 12 | 0.07 |
| 103 | Baghdad Teaching Hospital | 18 | 0.10 |
| 104 | Baxshin Hospital | 196 | 1.13 |
| 105 | Central Child Hospital | 35 | 0.20 |
| 106 | Child Protection Hospital | 33 | 0.19 |
| 107 | Emam Hussain Medical City | 76 | 0.44 |
| 108 | Especial Nursing Home Hospital | 36 | 0.21 |
| 109 | Fatmih Al-Zahraa Hospital | 23 | 0.13 |
| 110 | Gazi Al-Hariri Hospital | 23 | 0.13 |
| 111 | General Teaching Hospital | 76 | 0.44 |
| 112 | GIT Hospital | 28 | 0.16 |
| 113 | Ibn Al-Nafees Hospital | 42 | 0.24 |
| 114 | K1 Hospital | 27 | 0.16 |
| 115 | Kerbala Children Teaching Hospital | 25 | 0.14 |
| 116 | Kurdistan Center for Gastroenterology and Hepatology | 35 | 0.20 |
| 117 | Oncology Teaching Hospital | 26 | 0.15 |
| 118 | Shahid Tahir Ali Walli Bag | 34 | 0.20 |
| 119 | Shar Teaching Hospital | 280 | 1.62 |
| **Italy** (23) | | | |
| 120 | A.-O.-Bianchi-Melacrino-Morelli-Reggio-Calabria | 24 | 0.14 |
| 121 | A.O. Pugliese Ciaccio Catanzaro | 56 | 0.32 |
| 122 | A.O.U. Mater Domini Catanzaro | 18 | 0.10 |
| 123 | A.O.U.I. di Verona, Verona | 54 | 0.31 |
| 124 | ASL TO 3 (Local Health Unit 3 - Torino) | 27 | 0.16 |
| 125 | Institute-A.S.S.T.-Spedali-civili-Brescia | 68 | 0.39 |
| 126 | IRCCS Sacro Cuore Don Calabria Hospital Negrar, Verona | 16 | 0.09 |
| 127 | Ospedale Ca' Foncello di Treviso | 8 | 0.05 |
| 128 | P.O.-G.-Jazzolino-Vibo-Valentia | 64 | 0.37 |
| **Japan** (24) | | | |
| 129 | Hayama Heart Center | 16 | 0.09 |
| 130 | Kinan Hospital | 23 | 0.13 |
| 131 | Kyotango City Yasaka Hospital | 57 | 0.33 |
| 132 | Mie University Hospital | 24 | 0.14 |
| 133 | Nagasaki Yuuai Hospital | 11 | 0.06 |

| **Table S1: Global distribution of hospitals and healthcare workers included in the study (continued)** | | | |
| --- | --- | --- | --- |
| **Country/Territory**  **Hospital ID** | **Hospital name** | **No. HCWs** | **% of total** |

| 134 | National Center for Child Health and Development | 27 | 0.16 |
| --- | --- | --- | --- |
| 135 | Niigata Prefectural Kakizaki Hospital | 13 | 0.08 |
| 136 | Niigata Prefectural Sakamachi Hospital | 11 | 0.06 |
| 137 | Niigata Prefectural Tokamachi Hospital | 15 | 0.09 |
| 138 | Sado Municipal Ryotsu Hospital | 15 | 0.09 |
| 139 | Japan Self-Defense Forces Central Hospital | 10 | 0.06 |
| 140 | Shimane University Hospital | 10 | 0.06 |
| 141 | Shinshiro Municipal Hospital | 16 | 0.09 |
| 142 | Shonan Fujisawa Tokushukai Hospital | 11 | 0.06 |
| 143 | Shonan Kamakura General Hospital | 10 | 0.06 |
| 144 | Tokushima Prefectural Kaifu Hospital | 44 | 0.25 |
| 145 | Tokyo Women's Medical University Hospital | 50 | 0.29 |
| **Jordan** (25) | | | |
| 146 | Al Essra Hospital | 20 | 0.12 |
| 147 | Al Hikmeh Hospital | 19 | 0.11 |
| 148 | Irbid Speciality Hospital | 32 | 0.18 |
| 149 | King Abdullah University Hospital | 199 | 1.15 |
| 150 | King Hussein Cancer Centre | 22 | 0.13 |
| **Korea** (26) | | | |
| 151 | Korea University College of Medicine, Korea University Ansan Hospital | 21 | 0.12 |
| **Lebanon** (27) | | | |
| 152 | American University of Beirut Medical Center | 47 | 0.27 |
| **Libya** (28) | | | |
| 153 | Abo-sitta Hospital | 29 | 0.17 |
| 154 | Tripoli Medical Center | 54 | 0.31 |
| 155 | Tripoli Medical Hospital | 27 | 0.16 |
| **Malaysia** (29) | | | |
| 156 | Hospital Canselor Tuanku Muhriz (Kebangsaan Medical Centre) | 278 | 1.61 |
| 157 | Hospital Pengajar Universiti Putra Malaysia (HPUPM) | 38 | 0.22 |
| 158 | Public Health Clinic-Duchess of Kent Hospital | 28 | 0.16 |
| 159 | Pusat Kesihatan Universiti, Universiti Putra Malaysia | 69 | 0.40 |
| 160 | Queen Elizabeth 1 Hospital | 24 | 0.14 |
| 161 | Queen Elizabeth 2 Hospital | 10 | 0.06 |
| 162 | University Malaya Medical Center | 40 | 0.23 |
| 163 | Universiti Teknologi MARA | 110 | 0.64 |
| **Mexico** (30) | | | |
| 164 | General Hospital Dr Nicolas San Juan | 52 | 0.30 |
| 165 | Mother & Child Institute; Mexico State Gynecology & Obstetrics Hospital | 100 | 0.58 |

| **Table S1: Global distribution of hospitals and healthcare workers included in the study (continued)** | | | |
| --- | --- | --- | --- |
| **Country/Territory**  **Hospital ID** | **Hospital name** | **No. HCWs** | **% of total** |

| **Morocco** (31) | | | |
| --- | --- | --- | --- |
| 166 | Ibn Sina University Hospital | 60 | 0.35 |
| 167 | Marrakech Mohammed VI Hospital | 40 | 0.23 |
| 168 | Mohammed V Military Hospital | 28 | 0.16 |
| **Nepal** (32) | | | |
| 169 | B.P. Koirala Institute of Health Science | 104 | 0.60 |
| 170 | Bharatpur Hospital | 87 | 0.50 |
| 171 | Bheri zonal Hospital | 30 | 0.17 |
| 172 | Bir Hospital | 84 | 0.49 |
| 173 | Civil Service Hospital, Baneshwor | 89 | 0.51 |
| 174 | College of Medical Sciences | 51 | 0.29 |
| 175 | Manipal Teaching Hospital | 94 | 0.54 |
| 176 | Manmohan Memorial Teaching Hospital | 33 | 0.19 |
| 177 | Parkland Hospital | 26 | 0.15 |
| 178 | Sukraraj Tropical and Infectious Diseases Hospital | 73 | 0.42 |
| 179 | Tribhuvan University, Teaching Hospital | 122 | 0.71 |
| **Netherlands** (22) | | | |
| 180 | Wilhelmina Children's Hospital Utrecht University | 9 | 0.05 |
| **New Zealand** (34) | | | |
| 181 | Christchurch Hospital | 158 | 0.91 |
| **Nigeria** (35) | | | |
| 182 | ABUAD Multisystem Hospital | 28 | 0.16 |
| 183 | Abubakar Tafawa Balewa University Clinic | 17 | 0.10 |
| 184 | Abubakar Tafawa Balewa University Teaching Hospital | 15 | 0.09 |
| 185 | Almanzoor Hospital Bauchi | 15 | 0.09 |
| 186 | Ekiti State University Teaching Hospital | 28 | 0.16 |
| 187 | Federal Medical Centre Yola | 28 | 0.16 |
| 188 | Federal Medical Centre, Ebute Metta | 119 | 0.69 |
| 189 | Federal Teaching Hospital, Gombe State | 42 | 0.24 |
| 190 | Federal Teaching Hospital, Ido-Ekiti | 32 | 0.18 |
| 191 | General Hospital Gamawa, Bauchi State | 11 | 0.06 |
| 192 | General Hospital Ganye | 10 | 0.06 |
| 193 | General Hospital Hong | 19 | 0.11 |
| 194 | General Hospital Michika | 8 | 0.05 |
| 195 | General Hospital Mubi | 8 | 0.05 |
| 196 | General Hospital Numan | 8 | 0.05 |
| 197 | Hasiya Bayero Paediatric Hospital | 35 | 0.20 |
| 198 | Infectious Disease Hospital Bayara Bauchi | 10 | 0.06 |
| 199 | Infectious Disease Hospital, Kano, Nigeria | 29 | 0.17 |

| **Table S1: Global distribution of hospitals and healthcare workers included in the study (continued)** | | | |
| --- | --- | --- | --- |
| **Country/Territory**  **Hospital ID** | **Hospital name** | **No. HCWs** | **% of total** |

| 200 | Jos University Teaching Hospital | 36 | 0.21 |
| --- | --- | --- | --- |
| 201 | Lagos State University Teaching Hospital | 13 | 0.08 |
| 202 | Lagos University Teaching Hospital | 43 | 0.25 |
| 203 | Muhammad Abdullahi Wase Teaching Hospital | 57 | 0.33 |
| 204 | Murtala Muhammad Specialist Hospital | 45 | 0.26 |
| 205 | Nigerian Institute of Medical Research | 15 | 0.09 |
| 206 | Olabisi Onabanjo University Teaching Hospital | 41 | 0.24 |
| 207 | Primary Healthcare Center Kofar Ran Bauchi | 15 | 0.09 |
| 208 | Specialist Hospital Yola | 19 | 0.11 |
| 209 | State Specialist Hospital Bauchi | 16 | 0.09 |
| 210 | Women and Children Hospital Bauchi | 14 | 0.08 |
| **Pakistan** (36) | | | |
| 211 | Aga Khan University | 19 | 0.11 |
| 212 | Dow University Hospital | 27 | 0.16 |
| 213 | Dr. Ruth Pfau Civil Hospital, Karachi | 72 | 0.42 |
| 214 | Federal General Hospital | 31 | 0.18 |
| 215 | Federal General Polyclinic Services Hospital | 14 | 0.08 |
| 216 | Lady Reading Hospital | 121 | 0.70 |
| 217 | National Institute of Rehabilitation Medicine | 8 | 0.05 |
| 218 | Pakistan Institute of Medical Sciences | 21 | 0.12 |
| 219 | Rahim Hospital | 19 | 0.11 |
| 220 | Services Hospital Lahore | 15 | 0.09 |
| **Palestine** (37) | | | |
| 221 | Abu Yousef Al-Najjar | 18 | 0.10 |
| 222 | Al-Aqsa Martyrs Hospital | 74 | 0.43 |
| 223 | Al-Razi Hospital | 18 | 0.10 |
| 224 | Al-Remal Health Center | 22 | 0.13 |
| 225 | Al-Shifa Hospital Compound | 92 | 0.53 |
| 226 | Al-Zakat Hospital | 15 | 0.09 |
| 227 | An-Najah National University Hospital | 16 | 0.09 |
| 228 | Beit Hanoun Hospital | 29 | 0.17 |
| 229 | European Hospital Gaza | 86 | 0.50 |
| 230 | Indonesian Hospital | 55 | 0.32 |
| 231 | Istishari Arab Hospital | 19 | 0.11 |
| 232 | Kamal Adwan Hospital | 43 | 0.25 |
| 233 | Nasser Medical Complex | 88 | 0.51 |
| 234 | Specialized Arab Hospital | 17 | 0.10 |

| **Table S1: Global distribution of hospitals and healthcare workers included in the study (continued)** | | | |
| --- | --- | --- | --- |
| **Country/Territory**  **Hospital ID** | **Hospital name** | **No. HCWs** | **% of total** |

| **Paraguay** (38) | | | |
| --- | --- | --- | --- |
| 235 | Regional Hospital of Coronel Oviedo – Paraguay | 18 | 0.10 |
| **Philippines** (39) | | | |
| 236 | Calauag St. Peter General Hospital | 31 | 0.18 |
| 237 | Garcia Memorial Provincial Hospital | 17 | 0.10 |
| 238 | Gumaca District Hospital | 27 | 0.16 |
| 239 | Holy Rosary Hospital | 26 | 0.15 |
| 240 | Laguna Medical Center | 20 | 0.12 |
| 241 | Las Pinas Doctors Hospital | 39 | 0.23 |
| 242 | Lopez Saint Jude General Hospital | 23 | 0.13 |
| 243 | Magsaysay Memorial District Hospital | 25 | 0.14 |
| 244 | ManilaMed Medical Center Manila | 43 | 0.25 |
| 245 | Mary Chiles General Hospital | 15 | 0.09 |
| 246 | Medical Center Parañaque | 16 | 0.09 |
| 247 | Novaliches District Hospital | 24 | 0.14 |
| 248 | Nuestra Senora de los Angeles General Hospital | 30 | 0.17 |
| 249 | Ospital ng Maynila Medical Center | 53 | 0.31 |
| 250 | Pasig City General Hospital | 12 | 0.07 |
| 251 | Philippines General Hospital | 49 | 0.28 |
| 252 | RAKKK Prophet Medical Center Inc. | 31 | 0.18 |
| 253 | San Diego de Alcala General Hospital | 23 | 0.13 |
| **Russia** (40) | | | |
| 254 | Moscow City Infectious Hospital No 2 | 12 | 0.07 |
| 255 | Vladimir City Emergency Hospital | 8 | 0.05 |
| **Serbia** (41) | | | |
| 256 | Clinical Center of Kragujevac | 39 | 0.23 |
| 257 | General (Regional) Hospital of Kosovska Mitrovica | 14 | 0.08 |
| **Somalia** (42) | | | |
| 258 | Somali Sudanese Specialized Hospital | 76 | 0.44 |
| **South Africa** (43) | | | |
| 259 | Kalafong Provincial Tertiary Hospital | 103 | 0.60 |
| 260 | Pretoria West Hospital | 10 | 0.06 |
| 261 | Tshwane District Hospital | 19 | 0.11 |
| **Spain** (44) | | | |
| 262 | Clinic of Barcelona | 57 | 0.33 |
| 263 | Hospital Sant Joan de Deu | 12 | 0.07 |
| 264 | Hospital Universitario Vall d'Hebron | 51 | 0.29 |
| 265 | Puerta del Mar University Hospital | 26 | 0.15 |

| **Table S1: Global distribution of hospitals and healthcare workers included in the study (continued)** | | | |
| --- | --- | --- | --- |
| **Country/Territory**  **Hospital ID** | **Hospital name** | **No. HCWs** | **% of total** |

| **Sri Lanka** (45) | | | |
| --- | --- | --- | --- |
| 266 | District Base Hospital-Teldeniya | 12 | 0.07 |
| 267 | District General Hospital-Nuwara Eliya | 17 | 0.10 |
| 268 | District General Hospital-Trincomalee | 15 | 0.09 |
| 269 | Faculty of Medicine, Eastern University of Sri Lanka | 21 | 0.12 |
| 270 | National Hospital-Kandy | 25 | 0.14 |
| 271 | SBSCH | 14 | 0.08 |
| 272 | Teaching (General) Hospital-Peradeniya | 16 | 0.09 |
| 273 | Teaching Hospital Anuradhapura | 57 | 0.33 |
| **Sudan** (46) | | | |
| 274 | Ahmed Gassim Hospital | 20 | 0.12 |
| 275 | Al Faisal Specialist Hospital | 12 | 0.07 |
| 276 | Al Fouad Specialized Hospital | 34 | 0.20 |
| 277 | Al Rayhan Hospital | 12 | 0.07 |
| 278 | Al Shab Teaching Hospital | 9 | 0.05 |
| 279 | Almonawara Special Hospital | 16 | 0.09 |
| 280 | Alrwada Hospital | 24 | 0.14 |
| 281 | Asia Hospital | 20 | 0.12 |
| 282 | Bahri Teaching Hospital | 15 | 0.09 |
| 283 | Ed Dueim Teaching Hospital | 22 | 0.13 |
| 284 | El Obeid Teaching Hospital | 14 | 0.08 |
| 285 | El-Ban Jadeed Teaching Hospital | 17 | 0.10 |
| 286 | Elshiekh Hospital | 11 | 0.06 |
| 287 | Gezira Center for Pediatric Surgery | 20 | 0.12 |
| 288 | Haj Alsafai Hospital | 28 | 0.16 |
| 289 | Imperial Hospital | 11 | 0.06 |
| 290 | Kassala Teaching Hospital | 18 | 0.10 |
| 291 | Noon Hospital | 10 | 0.06 |
| 292 | Ombada Teaching Hospital | 25 | 0.14 |
| 293 | Omdurman Teaching Hospital | 40 | 0.23 |
| 294 | Soba University Hospital | 12 | 0.07 |
| 295 | Tuga Hospital | 18 | 0.10 |
| 296 | Turkish Hospital | 16 | 0.09 |
| 297 | Wad Medani General Pediatric Hospital | 30 | 0.17 |
| 298 | Wad Medani Obse & Gynae | 27 | 0.16 |
| 299 | Wad Medani Teaching Hospital | 25 | 0.14 |
| 300 | Yetbshroon Hospital | 11 | 0.06 |

| **Table S1: Global distribution of hospitals and healthcare workers included in the study (continued)** | | | |
| --- | --- | --- | --- |
| **Country/Territory**  **Hospital ID** | **Hospital name** | **No. HCWs** | **% of total** |

| **Syria** (47) | | | |
| --- | --- | --- | --- |
| 301 | Al-Assad Hospital | 39 | 0.23 |
| 302 | Al-Mouwasat Hospital | 35 | 0.20 |
| 303 | Alzahrawi Hospital | 25 | 0.14 |
| 304 | Damascus Hospital | 64 | 0.37 |
| 305 | Ibn Alnafes Hospital | 45 | 0.26 |
| 306 | Kidney Surgical Hospital | 24 | 0.14 |
| 307 | Peronist Hospital | 30 | 0.17 |
| **Taiwan** (48) | | | |
| 308 | Dou-Liou branch, NCKUH | 17 | 0.10 |
| 309 | National Cheng Kung University Hospital | 305 | 1.76 |
| 310 | Tainan Hospital | 9 | 0.05 |
| **Tanzania** (49) | | | |
| 311 | Kilimnajro Christian Medical Center | 27 | 0.16 |
| **Thailand** (50) | | | |
| 312 | Central Chest Institute of Thailand | 122 | 0.71 |
| 313 | King Chulalongkorn Memorial Hospital | 152 | 0.88 |
| 314 | Mae Sai Hospital | 239 | 1.38 |
| 315 | Maharaj Nakorn Chiang Mai Hospital | 167 | 0.97 |
| 316 | Nakornping Hospital | 45 | 0.26 |
| 317 | Sansai Hospital | 158 | 0.91 |
| **Trinidad & Tobago** (51) | | | |
| 318 | Sangre Grande Hospital | 116 | 0.67 |
| **Tunisia** (52) | | | |
| 319 | Ali Trad Basic Health Center | 18 | 0.10 |
| 320 | El Omrane University Hospital of Monastir | 9 | 0.05 |
| 321 | Enfidha Circonscription Hospital | 14 | 0.08 |
| 322 | Hedi Chaker University Hospital | 32 | 0.18 |
| 323 | Hospital of Interventional Forses | 56 | 0.32 |
| 324 | La Rabta University Hospital | 19 | 0.11 |
| 325 | Principal Military Hospital of Instruction of Tunis | 25 | 0.14 |
| 326 | Psychiatric University Hospital of Razi | 17 | 0.10 |
| 327 | Sahloul University Hospital | 25 | 0.14 |
| 328 | Sidi Hassine Basic Health Center | 14 | 0.08 |
| 329 | University Hospital of Ben Arous, Yasminette | 21 | 0.12 |
| **United Arab Emirates** (53) | | | |
| 330 | University Hospital Sharjah | 28 | 0.16 |

| **Table S1: Global distribution of hospitals and healthcare workers included in the study (continued)** | | | |
| --- | --- | --- | --- |
| **Country/Territory**  **Hospital ID** | **Hospital name** | **No. HCWs** | **% of total** |

| **Ukraine** (54) | | | |
| --- | --- | --- | --- |
| 331 | Bila Tserkva City Hospital No 2 | 42 | 0.24 |
| 332 | Kyiv City Clinical Hospital No 9 | 12 | 0.07 |
| 333 | Oleksandriia Central City Hospital No 1 | 29 | 0.17 |
| 334 | Oleksandriia Central District Hospital | 29 | 0.17 |
| 335 | Oleksandriia Primary Healthcare Center | 26 | 0.15 |
| 336 | Shalimov National Institute of Surgery and Transplantology | 11 | 0.06 |
| 337 | St. Paraskeva Medical Center | 20 | 0.12 |
| **United States** (55) | | | |
| 338 | Hospital of the University of Pennsylvania | 70 | 0.40 |
| 339 | MedStar, Washington Hospital Center | 14 | 0.08 |
| 340 | Norwalk Hospital | 37 | 0.21 |
| 341 | Penn Presbyterian Medical Center | 17 | 0.10 |
| 342 | Pennsylvania Hospital | 16 | 0.09 |
| **Vietnam** (56) | | | |
| 343 | 199 Hospital | 70 | 0.40 |
| 344 | Cao Van Chi Hospital | 26 | 0.15 |
| 345 | Children's Hospital 1 | 104 | 0.60 |
| 346 | Children's Hospital 2 | 252 | 1.46 |
| 347 | Cho Ray Hospital | 267 | 1.54 |
| 348 | City Children's Hospital | 183 | 1.06 |
| 349 | Danang Hospital | 164 | 0.95 |
| 350 | E Hospital | 104 | 0.60 |
| 351 | Family Hospital | 101 | 0.58 |
| 352 | FV Hospital | 76 | 0.44 |
| 353 | Gia Dinh People Hospital | 132 | 0.76 |
| 354 | Hue City Hospital | 26 | 0.15 |
| 355 | Hung Vuong Hospital | 30 | 0.17 |
| 356 | Military Hospital 211 | 206 | 1.19 |
| 357 | My Duc Mental Health Hospital | 20 | 0.12 |
| 358 | National Hospital of Traditional Medicine | 66 | 0.38 |
| 359 | Nghe An Northwest Regional Hospital | 263 | 1.52 |
| 360 | Nguyen Tri Phuong Hospital | 110 | 0.64 |
| 361 | Quang Nam Hospital for Women and Children | 54 | 0.31 |
| 362 | Quynh Phu General Hospital | 36 | 0.21 |
| 363 | Thanh Hoa General Hospital | 86 | 0.50 |
| 364 | Tien Giang Center General Hospital | 48 | 0.28 |
| 365 | Traditional Medicine Hospital | 53 | 0.31 |
| 366 | University Medical Center Campus No.1 and No.3 | 205 | 1.18 |

| **Table S1: Global distribution of hospitals and healthcare workers included in the study (continued)** | | | |
| --- | --- | --- | --- |
| **Country/Territory**  **Hospital ID** | **Hospital name** | **No. HCWs** | **% of total** |

| 367 | University Medical Center Campus No.2 | 89 | 0.51 |
| --- | --- | --- | --- |
| **Zambia** (57) | | | |
| 368 | Levy Mwanawasa General Hospital | 20 | 0.12 |
| 369 | Livingstone Central Hospital | 35 | 0.20 |
| 370 | Ndola Teaching Hospital | 17 | 0.10 |
| 371 | University Teaching Hospital - Zambia | 54 | 0.31 |

| **Table S2: List of hospitals that provided the Institutional Review Board approvals** | | |
| --- | --- | --- |
| **Country or territory** | **Hospital name** | **Name of IRB (English) / Name of IRB (as applicable)** |
| **Afghanistan** | Aliabd Teaching Hospital | Afghanistan National Charity Organization for Special Diseases |
|  | Blossom Health Care Center | Afghanistan National Charity Organization for Special Diseases |
|  | Jamhuriat Hospital | Afghanistan National Charity Organization for Special Diseases |
|  | Maiwand Teaching Hospital | Afghanistan National Charity Organization for Special Diseases |
|  | Rabia Balkhi Hospital | Afghanistan National Charity Organization for Special Diseases |
|  | Wazir Mohammad Akber Khan | Afghanistan National Charity Organization for Special Diseases |
| **Albania** | Gynecological Obstetric University Hospital "Ko‡o Gliozheni" | Hospital universitary obstetric gynecology "Koco Gliozheni" / Gynecological Obstetric University Hospital "Koço Gliozheni" |
| **Algeria** | Frantz Fanon Hospital Blida | Frantz Fanon Hospital Blida / UMC, CHU Blida, Frantz Fanon Hospital Blida |
| **Australia** | Westmead Hospital | WSLHD Human Research Ethics Commette |
| **Bangladesh** | Chittagong Medical College Hospital | Ethical Clearance of Chittagong Medical College |
|  | Mymensingh Medical College Hospital | Institutional Review Board Clearance of Mymensingh Medical College |
|  | Rajshahi Medical College Hospital | Ethical Review committee of Rajshahi Medical College |
|  | Sylhet MAG Osmani Medical College Hospital | Directorate General of Health Services, Mohakhali, Dhaka for Sylhet MAG Osmani Medical College hospital |
| **Brazil** | Hospital Regional Walter Alberto Pecoits OR Hospital Regional do Sudoeste do Paraná | Hospital Regional do Oeste do Paraná and Faculty of Medicine Committee / Pró-Reitoria de Pesquisa e Pós-Graduação Comitê de Ética em Pesquisa |
| **Cameroon** | Banyo District Hospital | Banyo District Hospital, Banyo, Cameroon |
|  | Centre Medical d'Arrondissement de Ngaoundal | Centre Medical d'Arrondissement de Ngaoundal, Ngaoundal, Cameroon |
|  | Regional Hospital of Buea | Regional Hospital of Buea |
| **Canada** | 1 | Queen's Universtity Health Science & Affilated Teaching Hospital Research Ethics Board |
|  | 2 | Queen's Universtity Health Science & Affilated Teaching Hospital Research Ethics Board |
|  | 3 | Queen's Universtity Health Science & Affilated Teaching Hospital Research Ethics Board |
|  | 4 | Queen's Universtity Health Science & Affilated Teaching Hospital Research Ethics Board |
| **Chile** | Hospital Hern n Henríquez Aravena | Dr. Hernán Henríquez Aravena Hospital / Hospital Dr. Hernán Henríquez Aravena |

| **Table S2: List of hospitals that provided the Institutional Review Board approvals (continued)** | | |
| --- | --- | --- |
| **Country or territory** | **Hospital name** | **Name of IRB (English) / Name of IRB (as applicable)** |
| **China** | Qilu Hospital of Shandong University | Shandong University Qilu Hospital |
|  | Shandong Provincial Qianfoshan Hospital | Shandong Provincial Qianfoshan Hospital, Shandong University |
|  | Shandong Provincial Third Hospital | Shandong Provincial Third Hospital |
|  | Subei People's Hospital | Subei People’s Hospital, Yangzhou Hospital of Traditional Chinese Medicine, Yangzhou University Hospital |
|  | The People's Hospital of Zouping City | The People's Hospital of Zouping City |
|  | The People's Hospital of Rizhao | The People's Hospital of Rizhao |
|  | The Second Hospital of Shandong University | Shandong University Second Hospital |
|  | Yan'an hospital affiliated to Kunming Medical University | Yan'an Hospital Kunming |
|  | Yangzhou First People's Hospital | Subei People’s Hospital, Yangzhou Hospital of Traditional Chinese Medicine, Yangzhou University Hospital |
|  | Yangzhou Hospital of Traditional Chinese Medicine | Subei People’s Hospital, Yangzhou Hospital of Traditional Chinese Medicine, Yangzhou University Hospital |
|  | Yidu Central Hospital of Weifang | Weifang Yidu Hospital |
| **Ecuador** | Clínica INFES | Clínica INFES |
|  | Hospital de Los Valles | Hospital de Los Valles |
|  | Hospital General Docente de Calderón | Hospital General Docente de Calderón |
|  | Hospital Vozandes | Hospital Vozandes Quito |
| **Egypt** | Abassia Fever Hospital | Abbassia Fever Hospital |
|  | Ain Shams University Hospitals (El-Demerdash) | Ain Shams Private Hospital |
|  | Ain Shams University Specialized Hospital | Ain Shams University Hospital |
|  | Al Hayat Hospital | Al Hayat Medical Services |
|  | Al-Azhar University Hospital | Al-Azhar University Hospital in Damietta |
|  | Al-Naql El-Bari Hospital | Alnaql Albari Parvati Hospital |
|  | Al-Sadr Hospital of Fayoum (chest hospital in fayoum) | Al- Sadr Hospital of Fayoum |
|  | Al-Safwa Specialist Hospital | Al- Safwa Specialist Hospital |
|  | Al-Zahraa Hospital | Al-Zahraa Hospital |
|  | Alexandria Main Hospital (Almiri) | Alexandria Main Hospital (Almiri), Alexandria, Egypt  لجنة الاخلاقيات الطبية كلية الطب البشري جامعة أسيو / |

| **Table S2: List of hospitals that provided the Institutional Review Board approvals (continued)** | | |
| --- | --- | --- |
| **Country or territory** | **Hospital name** | **Name of IRB (English) / Name of IRB (as applicable)** |
| **Egypt (continued)** | Alexandria Scientific Research Hospital | Alexandria Scientific Research Hospital |
|  | Assiut Main University Hospital | Assiut Main University Hospital, Assiut, Egypt |
|  | Assiut Pediatrics University Hospital | Assiut Pediatrics and Newborns Hospital, Assiut, Egypt |
|  | Aswan University Hospital | Aswan University Hospital |
|  | Atsa Central Hospital | General Hospital of Asta Fayoum |
|  | Burg El-Arab University Hospital | Burg El-Arab University Hospital, Alexandria, Egypt |
|  | Damietta Fever Hospital | Damietta Fever Hospital |
|  | El Shatby Hospital for Pediatrics and Newborns | El Shatby Hospital for Pediatrics and Newborns |
|  | El-Mowasah University Hospital | El-Mowasah University Hospital |
|  | El-Talaba University Hospital | El Talaba University Hospital, Assiut |
|  | Gerdo Primary Health Centre | Local Health Unite in Garedo el Fayoum |
|  | Internal Medicine University Hospital of Fayoum | Internal Medicine University Hospital of Fayoum |
|  | Manshe'at El-Bakry Hospital | Mansheat Bakry Hospital |
|  | Mostafa Hassan Teaching Hospital | Pediatric University Hospital of Fayoum |
|  | New Assuit University Hospital | New Assuit Hospital |
|  | Om El-Qosor University Hospital | Om El-Qosor University Hospital in Manfalut |
|  | Omooma Hospital | Omooma Private Hospital, Shebin El Koam, Menoufia, Egypt |
|  | Smouha University Hospital | Smouha University Hospital, Alexandria, Egypt |
|  | Tanta University Educational Hospital | Tanta Hospital |
|  | Zagazig University Hospital | Zagazig University Hospital |
| **Ethiopia** | Dessie Referral Hospital | Dessie Referral Hospital |
|  | Dil-chora | Chief Excutive Officer Dilchora Hospital Dire Dawa |
|  | Jimma Medical Center | Chief Clinical Director of Jimma Medical Center |
|  | Selam General Hospital | Selam General Hospital |
|  | Tikur Anbessa Specialized Hospital | Tikur Anbessa Specialized Hospital: Clinical service director of Tikur Anbessa Specialized Hospital |
|  | Yekatit-12 hospital | The Institutional Review Board of Yekatit-12 Hospital Medical College |
|  | Zewditu Memorial Hospital | Zewditu Memorial Hospital |
| **France** | Hospital Group Pellegrin Chu De Bordeaux | University Hospital of Bordeaux |

| **Table S2: List of hospitals that provided the Institutional Review Board approvals (continued)** | | |
| --- | --- | --- |
| **Country or territory** | **Hospital name** | **Name of IRB (English) / Name of IRB (as applicable)** |
| **France (continued)** | Piti‚ Salpetriere | France Society of Anaesthesiology, Resuscitation / Centre Hospitalier Universitaire de Bordeaux |
| **Germany** | University Hospital of Giessen | Ethics Committee at the Faculty of Medicine, Justus-Liebig University Giessen / Ethik-Kommission am Fachbereich Medizin, Justus-Liebig Universität Giessen |
| **Greece** | Athens Naval and Venterans Hospital | Athens Naval and Veterans Hospital |
| **Guatemala** | Liga Nacional contra el Cáncer INCAN | Liga Nacional Contra el Cáncer INCAN / Société Française d'Anesthésie et de Réanimation |
| **Hong Kong** | Pamela Youde Nethersole Eastern Hospital | Pamela Youde Nethersole Eastern Hospital |
|  | Queen Mary Hospital | Queen Mary Hospital |
| **India** | Bangalore Medical College and Research Institute | Bangalore Medical College & Research Institute |
|  | Computerized Pathology Lab | Computerised Pathology Lab |
|  | Indira Gandhi Institute of Dental Sciences, Kothamangalam, Kerala, India | Indira Gandhi Institute of Dental Science |
|  | RDT Hospital, Bathalapalli, AP, India | Director RTD Hospital Bathalaballi / RDT Hospital |
|  | Smt Kashibai Navale Medical College and General Hospital, Pune | Smt Kashibai Navale Medical College and General Hospital, Institutional Ethics Committee |
| **Indonesia** | Bangli Hospital-Bali | Komite Etik Penelitian Ketehatan |
|  | Bhayangkara Lemdiklat Polri-Jakarta | Komite Etik Penelitian Ketehatan |
|  | Panti Rapih Hospital, Yogyakarta | Komite Etik Penelitian Ketehatan |
|  | Sungai Dareh Hospital-West Sumatra | Komite Etik Penelitian Ketehatan |
|  | Yuliddin Away Hospital-Aceh | Komite Etik Penelitian Ketehatan |
| **Iran** | Amir al Momenin | Arak University of Medical Science Research Ethics Certificate |
|  | Ayatollah Khansari | Arak University of Medical Science Research Ethics Certificate |
|  | Valiasr Hospital | Arak University of Medical Science Research Ethics Certificate |
| **Iraq** | Al Hussain Teaching Hospital | Al-Hussein Teaching Hospital |
|  | AL Rometha Hospital | Al-Romatha General Hospital |
|  | Al wasiti Educational Hospital | Al-Waseti Hospital |
|  | Al-Hilla Teaching Hospital | Al Hilla Teaching Hospital |
|  | Al-Hindiya General Hospital | Al Hindiya General Hospital |
|  | Al-Kadhumain Teaching Hospital | Al-Kadhumain Teaching Hospital |
| **Table S2: List of hospitals that provided the Institutional Review Board approvals (continued)** | | |
| **Country or territory** | **Hospital name** | **Name of IRB (English) / Name of IRB (as applicable)** |
| **Iraq (continued)** | Al-Khudhr General Hospital | Al-Khudhr General Hospital |
|  | Al-Yarmuk Teaching Hospital | Al-Yarmuk Teaching Hospital |
|  | Baghdad Teaching Hospital | Baghdad Teaching Hospital / Liga Nacional Contra el Cáncer |
|  | Baxshin Hospital | Baxshin Private Hospital |
|  | Central Child Hospital | Central Child Hospital |
|  | Child Protection Hospital | Child Protection Hospital |
|  | Emam Hussain Medical City | Emam Hussain Medical City |
|  | Especial Nursing Home Hospital | Especial Nursing Home Hospital |
|  | Fatmih Al-Zahraa | Fatmih Al-Zahraa Maternity and Children Teaching Hospital |
|  | Gazi Al-Hariri Hospital | Gazi Al-Hariri Hospital |
|  | General Teaching Hospital (Shaheed Dr Hemn) | General Teaching Hospital of (Shahid Dr Hemn) |
|  | GIT Hospital | Digestive System Teaching Hospital |
|  | Ibn Al-Nafees | Ibn Al-Nafees Teaching Hospital |
|  | K1 Hospital | K1 Hospital (Kaywan Hospital) |
|  | Kerbala Pediatric Teaching Hospital | Kerbala Pediatric Teaching Hospital |
|  | Kurdistan Center for Gastroenetrology and Hepatology | Kurdistan Center for Gastroenterology and Hepatology |
|  | Oncology Teaching hospital | Oncology Teaching Hospital |
|  | Shahid Tahir Ali Walli Bag | Shahid Tahir Ali Walli Bag Hospital |
|  | Shar teaching hospital | Shar Teaching Hospital |
| **Italy** | A.O. Bianchi-Melacrino-Morelli-Reggio-Calabria | Bianchi-Melacrino-Morelli Hospital / Grande Osspedale Metropolitano (Bianchi-Melacrino-Morelli) |
|  | A.O. Pugliese Ciaccio Catanzaro | Pugliese Ciaccio Hospital / Azienda Ospedaliera "Pugliese Ciaccio" |
|  | A.O.U. Mater Domini Catanzaro | Humanitas Mater Domini Hospital / Azienda Ospedaliero-Universitaria "Mater Domini" |
|  | A.O.U.I. di Verona, Verona | University Hospital of Verona / Azienda Ospedaliera Universitaria Integrata Verona |
|  | ASL TO 3 (Local Health Unit 3, Torino) | ASL TO 3 (Local Health Unit 3, Torino) |
|  | Institute-A.S.S.T. Spedali-Civili-Brescia | Spedali Civili Hospital / A.S.S.T. Spedali Civili |
|  | IRCCS Sacro Cuore Don Calabria Hospital Negrar, Verona | Sacro Cuore Don Calabria Hospital / Istituto di Ricovero e Cura a Carattere Scientifico, Sacro Cuore - Don Calabria |
|  | Ospedale Ca' Foncello di Treviso | Ca' Foncello Hospital of Treviso / Regione Del Veneto, Ospedaliero di Treviso |
|  | P.O.G. Jazzolino-Vibo-Valentia | G. Jazzolino Hospital / Azienda Sanitaria Provinciale Vibo Valentia |
| **Table S2: List of hospitals that provided the Institutional Review Board approvals (continued)** | | |
| **Country or territory** | **Hospital name** | **Name of IRB (English) / Name of IRB (as applicable)** |
| **Japan** | Hayama Heart Center registered as Kanagawa Shonan Tokushukai Hospital | Hayama Heart Center registered as Kanagawa Shonan Tokushukai Hospital |
|  | Kinan Hospital | Kinan Hospital |
|  | Kyotango City Yasaka Hospital | Kyotango City Yasaka Hospital |
|  | Mie University Hospital | Mie University Hospital |
|  | Nagasaki Yuuai Hospital | Nagasaki Yuuai Hospital |
|  | National Center for Child Health and Development | National Center for Child Health and Development |
|  | Niigata Prefeftural Kakizaki Hospital | Niigata Prefeftural Kakizaki Hospital |
|  | Niigata Prefeftural Sakamachi Hospital | Niigata Prefeftural Sakamachi Hospital |
|  | Niigata Prefeftural Tokamachi Hospital | Niigata Prefeftural Tokamachi Hospital |
|  | Sado Municipal Ryotsu Hospital | Sado Municipal Ryotsu Hospital |
|  | Self-Defence Forces Central Hospital | Self-Defence Forces Central Hospital |
|  | Shimane University Hospital | Shimane University Hospital |
|  | Shinshiro Municipal Hospital | Shinshiro Municipal Hospital |
|  | Shonan Fujisawa Tokushukai Hospital | Shonan Fujisawa Tokushukai Hospital |
|  | Shonan Kamakura General Hospital | Shonan Kamakura General Hospital |
|  | Tokushima Prefectural Kaifu Hospital | Tokushima Prefectural Kaifu Hospital |
|  | Tokyo Women's Medical University | Tokyo Women's Medical University |
| **Jordan** | Al Essra Hospital | Al- Isra Hospital |
|  | Al Hikmeh Hospital | Al-Hikmeh Hospital |
|  | Irbid Speciality Hospital | Irbid Specialty Hospital |
|  | King Abdullah University Hospital | King Abdullah University Hospital |
|  | King Hussein Cancer Centre | King Hussain Cancer Center |
| **Korea** | Korea University College of Medicine, Korea University Ansan Hospital | Korea University College of Medicine, Korea University Ansan Hospital |
| **Lebanon** | American University of Beirut Medical Center | Institutional Review Board American University of Beirut |
| **Libya** | Abo Sitta hospital | Abo Sitta Hospital |
|  | Tripoli Medical Center | Tripoli Medical Center |
|  | Tripoli Medical Hospital | Tripoli Central Hospital |

| **Table S2: List of hospitals that provided the Institutional Review Board approvals (continued)** | | |
| --- | --- | --- |
| **Country or territory** | **Hospital name** | **Name of IRB (English) / Name of IRB (as applicable)** |
| **Malaysia** | Hospital Canselor Tuanku Muhriz (University Kebangsaan Malaysia Medical Centre) | Research Ethics Committee, The National University of Malaysia |
|  | Hospital Pengajar Universiti Putra Malaysia | Ethics Committee for Research Involving Human Subjects - Universiti Putra Malaysia |
|  | Public Health Clinic-Duchess of Kent Hospital | Medical Research Ethics Committee, University Malaya Medical Center |
|  | Pusat Kesihatan Universiti Putra Malaysia | Ethics Committee for Research Involving Human Subjects - Universiti Putra Malaysia |
|  | Queen Elizabeth 1 Hospital | Medical Research Ethics Committee, University Malaya Medical Center |
|  | Queen Elizabeth 2 Hospital | Medical Research Ethics Committee, University Malaya Medical Center |
|  | Univeristy Malaya Medical Center (UMMC PPUM) | Medical Research Ethics Committee, University Malaya Medical Center |
|  | Universiti Teknologi MARA (UiTM) | UiTM Research Ethics Committee |
| **Mexico** | General Hospital Dr Nicolas San Juan | General Hospital Dr. Nicolas San Juan / Hospital General Doctor Nicolás San Juan |
|  | Mother and Child Instituite of Mexico State Gynecology and Obstetrics Hospital | Gynecology and Obstetrics Hospital, Mother and Child Institute of Mexico State / Instituto Materno Infantil del Estado de México, Hospital de Ginecología y Obstetricia |
| **Morocco** | Ibn Sina University Hospital | Director of Ibn Sina University Hospital / Directeur de l'hopital Ibn Sina |
|  | Marrakech Mohammed VI Hospital | Director of Mohammed IV Hospital-University Center / Directeur du Centre Hospitalo-Universitaire Mohammed IV |
|  | Mohammed V Military Hospital | Director of Mohammed IV Hospital-University Centerb |
| **Nepal** | B.P. Koirala Institute of Health Science | B.P. Koirala Institute of Health Sciences |
|  | Bharatpur Hospital | Bharatpur Hospital |
|  | Bheri Zonal Hospital | Bheri Hospital |
|  | Bir Hospital | Bir Hospital |
|  | Civil Service Hospital, Baneshwor | Civil Service Hospital / National IRB - Nepal Health Research Council (NHRC) |
|  | College of Medical Sciences | College of Medical Sciences - Teaching Hospital |
|  | Manipal Teaching Hospital | Manipal College of Medical Science Pokhara |
|  | Manmohan Memorial Teaching Hospital | Manmohan Memorial Medical College & Teaching Hospital |
|  | Parkland Hospital | Parkland Hospital |
|  | Sukraraj Tropical and Infectious Diseases Hospital | Sukraraj Tropical & Infectious Disease Hospital |
|  | Tribhuvan University Teaching Hospital | Tribhuvan University Teaching Hospital |
| **Table S2: List of hospitals that provided the Institutional Review Board approvals (continued)** | | |
| **Country or territory** | **Hospital name** | **Name of IRB (English) / Name of IRB (as applicable)** |
| **Netherlands** | Wilhelmina Children's Hospital Utrecht University | IRB exemption |
| **New Zealand** | Christchurch Hospital | Ethics Committee University of Otago |
| **Nigeria** | ABUAD Multisystem Hospital | ABUAD Multisystem Hospital |
|  | Abubakar Tafawa Balewa University Teaching Hospital | Abubakar Tafawa Balewa University Teaching Hospital |
|  | Almanzoor Hospital Bauchi | Almanzoor Hospital Bauchi |
|  | Ekiti State University Teaching Hospital | Ekiti State University Teaching Hospital |
|  | Federal Medical Centre Yola | Federal Medical Centre Yola |
|  | Fedral Medical Centre, Ebute Metta | Fedral Medical Centre, Ebute Metta |
|  | Federal Teaching Hospital, Gombe | Federal Teaching Hospital, Gombe |
|  | Federal Teaching Hospital, Ido-Ekiti | Federal Teaching Hospital, Ido-Ekiti |
|  | General Hospital Gamawa Bauchi State | General Hospital Gamawa Bauchi State |
|  | General Hospital Ganye | General Hospital Ganye |
|  | General Hospital Hong | General Hospital Hong |
|  | General Hospital Michika | General Hospital Michika |
|  | General Hospital Mubi | General Hospital Mubi |
|  | General Hospital Numan | General Hospital Numan |
|  | Hasiya Bayero Paediatric Hospital | Hasiya Bayero Paediatric Hospital |
|  | Infectious Disease Hospital Bayara Bauchi | Infectious Disease Hospital Bayara Bauchi |
|  | Infectious Disease Hospital, Kano, Nigeria | Infectious Disease Hospital, Kano, Nigeria |
|  | Jos University teaching Hospital | Jos University Teaching Hospital |
|  | Lagos State University Teaching Hospital (Lasuth), Ikeja | Lagos State University Teaching Hospital (Lasuth), Ikeja |
|  | Lagos University Teaching Hospital | Lagos University Teaching Hospital |
|  | Muhammad Abdullahi Wase Teaching Hospital | Muhammad Abdullahi Wase Teaching Hospital |
|  | Murtala Muhammad Specialist Hospital | Murtala Muhammad Specialist Hospital |
|  | Nigerian Institute of Medical Research | Nigerian Institute of Medical Research |
|  | Olabisi Onabanjo University Teaching Hospital | Olabisi Onabanjo University Teaching Hospital |
|  | Primary Healthcare Center Kofar Ran Bauchi | Primary Healthcare Center Kofar Ran Bauchi |
| **Table S2: List of hospitals that provided the Institutional Review Board approvals (continued)** | | |
| **Country or territory** | **Hospital name** | **Name of IRB (English) / Name of IRB (as applicable)** |
| **Nigeria (continued)** | Specialsit Hospital Yola | Specialsit Hospital Yola |
|  | State Specialist Hospital Bauchi | State Specialist Hospital Bauchi |
|  | Women and Children Hospital Bauchi | Women and Children Hospital Bauchi |
| **Pakistan** | Agha Khan University | Agha Khan University |
|  | Dow University Hospital | Dow University Hospital |
|  | Dr. Ruth Pfau Civil Hospital, Karachi | Dr. Ruth Pfau Civil Hospital, Karachi |
|  | Federal General Hospital | Federal General Hospital |
|  | Federal General Polyclinic Services Hospital | Federal General Polyclinic Services Hospital |
|  | Lady Reading Hospital | Lady Reading Hospital |
|  | National Institute of Rehabilitation medicine | National Institute of Rehabilitation medicine |
|  | Pakistan Institute of Medical Sciences | Pakistan Institute of Medical Sciences |
|  | Rahim Hospital | Rahim Hospital |
|  | Services Hospital Lahore | Services Hospital Lahore |
| **Palestine** | Abu Yousef Al-Najjar | General Adminstration of Manupower Development - Ministry of Health / الادراة العامة لتنمية القوى البشرية - وزارة الصحة |
|  | Al-Aqsa Martyrs Hospital | General Adminstration of Manupower Development - Ministry of Health /  الادراة العامة لتنمية القوى البشرية - وزارة الصحة |
|  | Al-Razi Hospital | Al-Razi Hospital Director Board /  مجلس ادراة مستشفى الرازي |
|  | Al-Remal Medical Hospital | General Adminstration of Manupower Development - Ministry of Health /  الادراة العامة لتنمية القوى البشرية - وزارة الصحة |
|  | Al-Shifa Hospital Compound | General Adminstration of Manupower Development - Ministry of Health /  الادراة العامة لتنمية القوى البشرية - وزارة الصحة |
|  | Al-Zakat Hospital | Al-Zakat Hospital Director Board /  مجلس ادراة مستشفى الزكاة |
|  | An-Najah National University Hospital | An-Najah National University Hospital Director Board /  مجلس ادراة مستشفى النجاح الوطني |
|  | Beit Hanoun Hospital | General Adminstration of Manupower Development - Ministry of Health /  الادراة العامة لتنمية القوى البشرية - وزارة الصحة |
|  | European Hospital Gaza | General Adminstration of Manupower Development - Ministry of Health /  الادراة العامة لتنمية القوى البشرية - وزارة الصحة |
|  | Indonesian Hospital | General Adminstration of Manupower Development - Ministry of Health /  الادراة العامة لتنمية القوى البشرية - وزارة الصحة |
| **Table S2: List of hospitals that provided the Institutional Review Board approvals (continued)** | | |
| **Country or territory** | **Hospital name** | **Name of IRB (English) / Name of IRB (as applicable)** |
| **Palestine (continued)** | Istishari Arab Hospital | Istishari Arab Hospital Director Board /  مجلس ادراة المستشفى الاستشاري العربي |
|  | Kamal Adwan Hospital | General Adminstration of Manupower Development - Ministry of Health /  الادراة العامة لتنمية القوى البشرية - وزارة الصحة |
|  | Nasser Medical Complex | General Adminstration of Manupower Development - Ministry of Health /  الادراة العامة لتنمية القوى البشرية - وزارة الصحة |
|  | Specialized Arab Hospital | Specialized Arab Hospital Director Board /  مجلس ادراة المستشفى العربي التخصصي |
| **Paraguay** | Regional Hospital of Coronel Oviedo - Paraguay | Regional Hospital of Coronel Oviedo – Paraguay |
| **Philippines** | Calauag St. Peter General Hospital | Single Joint Research Ethics Board |
|  | Garcia Memorial Provincial Hospital | Single Joint Research Ethics Board |
|  | Gumaca District Hospital | Single Joint Research Ethics Board |
|  | Holy Rosary Hospital | Single Joint Research Ethics Board |
|  | Laguna Medical Center | Single Joint Research Ethics Board |
|  | Las Pinas Doctors Hospital | Single Joint Research Ethics Board |
|  | Lopez Saint Jude General Hospital | Single Joint Research Ethics Board |
|  | Magsaysay Memorial District Hospital | Single Joint Research Ethics Board |
|  | ManilaMed Medical Center Manila | Single Joint Research Ethics Board |
|  | Mary Chiles General Hospital | Single Joint Research Ethics Board |
|  | Medical Center Para¤aque | Single Joint Research Ethics Board |
|  | Novaliches District Hospital | Single Joint Research Ethics Board |
|  | Nuestra Senora De los Angeles General Hospital | Single Joint Research Ethics Board |
|  | Ospital ng Maynila Medical Center | Single Joint Research Ethics Board |
|  | Pasig City General Hospital | Single Joint Research Ethics Board |
|  | Philippines General Hospital | Single Joint Research Ethics Board |
|  | RAKKK Prophet Medical Center Inc. | Single Joint Research Ethics Board |
|  | San Diego de Alcala General Hospital | Single Joint Research Ethics Board |
| **Russia** | Moscow City Infectous Hospital No 2 | Infectious Diseases Clinical Hospital №2 of Moscow /  Инфекционная клиническая больница №2 города Москвы |
|  | Vladimir City Emergency Hospital | Chairman of Surgery Vladimir City Emergency Hospital |
| **Table S2: List of hospitals that provided the Institutional Review Board approvals (continued)** | | |
| **Country or territory** | **Hospital name** | **Name of IRB (English) / Name of IRB (as applicable)** |
| **Serbia** | Clinical Center of Kragujevac | Ethical Committee Clinical Centre Kragujevac |
|  | General (Regional) Hospital of Kosovska Mitrovica | Institutional Review Board Rocklnad Community College |
| **Somalia** | Somali Sudanese Specialized Hospital | Somali Sudanese Specialist Hospital |
| **South Africa** | Kalafong Provincial Tertiary Hospital | Kalafong Provincial Tertiary Hospital |
|  | Pretoria West Hospital | Pretoria West Hospital |
|  | Tshwane District Hospital | Tshwane District Hospital |
| **Spain** | Clinic of Barcelona | Head of Hospital Clínic Maternitat, Hospital Clinic of Barcelona |
|  | Hospital Sant Joan de Deu | Head of Hospital Sant Joan de Dèu |
|  | Hospital Universitario Vall d'Hebron | Ethics Committee for Research with Medicines of the University Hospital Vall d'Hebron |
|  | Puerta del Mar University Hospital | The Institutional Ethics Committee of Cádiz |
| **Sri Lanka** | District Base Hospital-Teldeniya | Deputy Provincial Director of Health Services Central Province |
|  | District General Hospital-Nuwara Eliya | Deputy General Hospital Nuwara Eliva |
|  | District General Hospital-Trincomalee | Director District General Hospital Trincomalee |
|  | Faculty of Medicine, Eastern University of Sri Lanka | Dean Faculty of Health-care Sciences? |
|  | National Hospital-Kandy | Director National Hospital Kandy |
|  | Sirimavo Bandaranaike Speacialized Children Hospital | Director Sirimavo Bandaranaike Speacialized Children Hospital Peradeniya |
|  | Teaching (General) Hospital-Peradeniya | Deputy Director General Hospital (Teaching) Peradeniya |
|  | Teaching Hospital Anuradhapura | Director of Anuradhapura Teaching Hospital |
| **Sudan** | Ahmed Gassim Hospital | National Health Research Ethics Committee, Federal Ministry of Health |
|  | Al Faisal Specialist Hospital | National Health Research Ethics Committee, Federal Ministry of Health |
|  | Al Fouad Specialized Hospital | National Health Research Ethics Committee, Federal Ministry of Health |
|  | Al Rayhan Hospital | National Health Research Ethics Committee, Federal Ministry of Health |
|  | Al Shab Teaching Hospital | National Health Research Ethics Committee, Federal Ministry of Health |
|  | AlmonawaraSspecial Hospital | National Health Research Ethics Committee, Federal Ministry of Health |
| **Table S2: List of hospitals that provided the Institutional Review Board approvals (continued)** | | |
| **Country or territory** | **Hospital name** | **Name of IRB (English) / Name of IRB (as applicable)** |
| **Sudan (continued)** | Alrwada Hospital | National Health Research Ethics Committee, Federal Ministry of Health |
|  | Asia Hospital | National Health Research Ethics Committee, Federal Ministry of Health |
|  | Bahri Teaching Hospital | National Health Research Ethics Committee, Federal Ministry of Health |
|  | Ed Dueim Teaching Hospital | National Health Research Ethics Committee, Federal Ministry of Health |
|  | El Obeid Teaching Hospital | National Health Research Ethics Committee, Federal Ministry of Health |
|  | El-Ban Jadeed Teaching Hospital | National Health Research Ethics Committee, Federal Ministry of Health |
|  | Elshiekh Hospital | National Health Research Ethics Committee, Federal Ministry of Health |
|  | Gezira Center for Paediatric Surgery | National Health Research Ethics Committee, Federal Ministry of Health |
|  | Haj Alsafai Hospital | National Health Research Ethics Committee, Federal Ministry of Health |
|  | Imperial Hospital | National Health Research Ethics Committee, Federal Ministry of Health |
|  | Kassala Teaching Hospital | National Health Research Ethics Committee, Federal Ministry of Health |
|  | Noon Hospital | National Health Research Ethics Committee, Federal Ministry of Health |
|  | Ombada Teaching Hospital | National Health Research Ethics Committee, Federal Ministry of Health |
|  | Omdurman Teaching Hospital | National Health Research Ethics Committee, Federal Ministry of Health |
|  | Soba University Hospital | National Health Research Ethics Committee, Federal Ministry of Health |
|  | Tuga Hospital | National Health Research Ethics Committee, Federal Ministry of Health |
|  | Turkish Hospital | National Health Research Ethics Committee, Federal Ministry of Health |
|  | Wad Medani General Paediatric Hospital | National Health Research Ethics Committee, Federal Ministry of Health |
|  | Wad Madani Hospital for Obstetrics and Gynaecology | National Health Research Ethics Committee, Federal Ministry of Health |
|  | Wad Medani Teaching Hospital | National Health Research Ethics Committee, Federal Ministry of Health |
|  | Yetbshroon Hospital | National Health Research Ethics Committee, Federal Ministry of Health |
| **Syria** | Al-Assad Hospital | Al-Assad-Hospital |
|  | Al-Mouwasat Hospital | Al-Mouwasat-Hospital |
|  | Alzahrawi Hospital | Alzahrawi Hospital |
| **Table S2: List of hospitals that provided the Institutional Review Board approvals (continued)** | | |
| **Country or territory** | **Hospital name** | **Name of IRB (English) / Name of IRB (as applicable)** |
| **Syria (continued)** | Damascus Hospital | Damascus Hospital |
|  | Ibn Alnafes Hospital | Ibn Alnafes Hospital |
|  | Kidney Surgical Hospital | Kidney Surgical Hospital |
|  | Peronist Hospital | Peronist-Hospital |
| **Taiwan** | Dou-Liou Branch, National Cheng Kung Unviersity Hospital | Dou-Liou Branch, National Cheng Kung Unviersity Hospital |
|  | National Cheng Kung University Hospital | Institutional Review Board of National Cheng Kung University Hospital |
|  | Tainan Hospital | Tainan Hospital, Ministry of Health and Welfare |
| **Tanzania** | Kilimanjaro Christian Medical Center | Kilimanjaro Christian Medical Center |
| **Thailand** | Central Chest Institute of Thailand | Central Chest Institute of Thailand |
|  | King Chulalongkorn Memorial Hospital | Institutional Review Board - Chulalongkorn University |
|  | Mae Sai Hospital | Maesai Hospital |
|  | Maharaj nakorn Chiang Mai Hospital | Faculty of Medicine - Chiangmai University |
|  | Nakornping Hospital | Research Ethics Committee - Nakornping Hospital |
|  | Sansai Hospital | Sansai Hospital |
| **Trinidad &Tobago** | Sangre Grande Hospital | Eastern Regional Health Authority |
| **Tunisia** | Ali Trad Basic Health Center | Ali Trad Basic Health Center Mellassine |
|  | El Omrane University Hospital of Monastir | El Omrane University Hospital of Monastir |
|  | Enfidha Circonscription Hospital | Ennfidha Circonscription Hospital |
|  | Hedi Chaker University Hospital | Hedi Chaker University Hospital of Sfax |
|  | Hospital of Interventional Forses | Hospital of Interventional Forses |
|  | La Rabta University Hospital | Rabta University Hospital |
|  | Principal Military Hospital of Instruction of Tunis | Principal Military Hospital of Instruction of Tunis |
|  | Psychiatric University Hospital of Razi | Psychiatric University Hospital of Razi |
|  | Sahloul University Hospital | Sahloul University Hospital |
|  | Sidi Hassine Basic Health Center | Sidi Hassine Mellassine Basic Health Center |
|  | University Hospital of Ben Arous, Yasminette | University Hospital of Ben Arous, Yasminette |
| **United Arab Emirates** | University Hospital Sharjah | University Hospital Sharjah |
| **Ukraine** | Bila Tserkva City Hospital №2 | Bila Tserkva City Hospital №2 |
|  | Kyiv City Clinical Hospital №9 | Kyiv City Clinical Hospital №9 |
| **Table S2: List of hospitals that provided the Institutional Review Board approvals (continued)** | | |
| **Country or territory** | **Hospital name** | **Name of IRB (English) / Name of IRB (as applicable)** |
| **Ukraine (continued)** | Oleksandriia Central City Hospital №1 | Oleksandriia Central City Hospital №1 |
|  | Oleksandriia Central District Hospital | Oleksandriia Central District Hospital |
|  | Oleksandriia Primary Healthcare Center | Oleksandriia Primary Healthcare Center |
|  | Shalimov National Institute of Surgery and Transplantology | Shalimov National Institute of Surgery and Transplantology |
|  | St. Paraskeva Medical Center | St. Paraskeva Medical Center |
| **United States of America** | Hospital of the University of Pennsylvania | Hospital of the University of Pennsylvania |
|  | MedStar, Washington Hospital Center, Washington, DC | MedStar, Washington Hospital Center, Washington, DC |
|  | Norwalk Hospital | Norwalk Hospital |
|  | Penn Presbyterian Medical Center | Penn Presbyterian Medical Center |
|  | Pennyslvania Hospital | Pennyslvania Hospital |
| **Vietnam** | 199 Hospital | 199 Hospital |
|  | Cao Van Chi Hospital | Cao Van Chi Hospital |
|  | Children's Hospital 1 | Children's Hospital 1 |
|  | Children's Hospital 2 | Children's Hospital 2 |
|  | Cho Ray Hospital | Cho Ray Hospital |
|  | City Children's Hospital | City Children's Hospital |
|  | Danang Hospital | Danang Hospital |
|  | E Hospital | E Hospital |
|  | Family Hospital | Family Hospital |
|  | FV Hospital | FV Hospital |
|  | Gia Dinh People Hospital | Gia Dinh Hospital |
|  | Hue City Hospital | Hue City Hospital |
|  | Hung Vuong Hospital | Hung Vuong Hospital |
|  | Military Hospital 211 | Military Hospital 211 |
|  | My Duc Mental Health Hospital | My Duc Mental Health Hospital |
|  | National Hospital of Traditional Medicine | National Hospital of Traditional Medicine |
|  | Nghe An Northwest Regional Hospital | Nghe An Northwest Regional Hospital |
|  | Nguyen Tri Phuong Hospital | Nguyen Tri Phuong Hospital |
|  | Quang Nam Hospital for Women and Children | Quang Nam Hospital for Women and Children |
|  | Quynh Phu General Hospital | Quynh Phu General Hospital |
|  | Thanh Hoa General Hospital | Thanh Hoa General Hospital |
|  | Tien Giang Center General Hospital | Tien Giang Center General Hospital |
| **Table S2: List of hospitals that provided the Institutional Review Board approvals (continued)** | | |
| **Country or territory** | **Hospital name** | **Name of IRB (English) / Name of IRB (as applicable)** |
| **Vietnam (continued)** | Traditional Medicine Hospital | Traditional Medicine Hospital, Viet Nam Ministry of Public Security |
|  | University Medical Center Campus No.1 and No.3 | University Medical Center Campus No.1 and No.3 |
|  | University Medical Center Campus No.2 | University Medical Center Campus No.2 |
| **Zambia** | Levy Mwanawasa General Hospital | Levy Mwanawasa General Hospital |
|  | Livingstone Central Hospital | Livingstone Central Hospital |
|  | Ndola Teaching Hospital | Ndola Teaching Hospital |
|  | University Teaching Hospital - Zambia | University Teaching Hospital - Zambia |

| Table S3: Sociodemographic characteristics, work experience, workplace, and source of information and related attitudes of participating healthcare workers | |
| --- | --- |
| Characteristic | Participants, *n* (%) |
| Sociodemographic | |
| Continent (*n* = 17 302) | |
| Africa | 4,102 (23.7) |
| Asia | 11,065 (64.0) |
| Australia | 192 (1.1) |
| Europe | 959 (5.5) |
| North America | 541 (3.1) |
| South America | 443 (2.6) |
| Region (*n* = 17 302) | |
| East Asia & Pacific | 7,752 (44.8) |
| Europe & Central Asia | 959 (5.5) |
| Latin America & Caribbean | 595 (3.4) |
| Middle East & North Africa | 3,833 (22.2) |
| North America | 389 (2.2) |
| South Asia | 1,810 (10.5) |
| Sub-Saharan Africa | 1,964 (11.4) |
| Country income level (*n* = 17 302) | |
| High income | 2,161 (12.5) |
| Upper middle income | 6,234 (36.0) |
| Lower middle income | 7,461 (43.1) |
| Low income | 1,446 (8.4) |
| Work experience and workplace | |
| Level of hospital (*n* = 17 050) | |
| 1^st^ level | 1,678 (9.8) |
| 2^nd^ level | 3,948 (23.2) |
| 3^rd^ level | 11,424 (67.0) |
| Source of information and related attitudes | |
| Sources of information about COVID-19 (*n* = 17 192) | |
| Mainstream media | 13,659 (79.4) |
| Social network | 11,336 (65.9) |
| Government organization | 9,603 (55.9) |
| Colleagues/Your hospital | 7,423 (43.2) |
| Academic training courses | 4,382 (25.5) |
| Other | 197 (1.1) |

| **Table S4: Summaries of support, confidence, lift values for the generated association rules** | | | | | | |
| --- | --- | --- | --- | --- | --- | --- |
| Profession  [ID] | **Antecedent** {Q1: satisfaction with the current medical equipment for the management of COVID-19 outbreak, Q2: confidence in handling suspected COVID-19 patients} | **Consequent** {Q3: training for dealing  with COVID-19 patients} | | Support | Confidence | Lift |
| Doctor | | | | | | |
| [1] | {Q1=Very satisfied, Q2=To a great extent} | => | {Q3=Yes} | 0.028 | 0.873 | 2.681 |
| [2] | {Q1=Satisfied, Q2=To a great extent} | => | {Q3=Yes} | 0.029 | 0.865 | 2.658 |
| [3] | {Q1=Unsatisfied, Q2=Not at all} | => | {Q3=No} | 0.066 | 0.932 | 1.382 |
| [4] | {Q1=Very unsatisfied, Q2=Not at all} | => | {Q3=No} | 0.048 | 0.912 | 1.353 |
| [5] | {Q1=Neutral, Q2=Not at all} | => | {Q3=No} | 0.030 | 0.906 | 1.344 |
| [6] | {Q1=Very unsatisfied, Q2=To a little extent} | => | {Q3=No} | 0.033 | 0.900 | 1.335 |
| [7] | {Q1=Satisfied, Q2=To a little extent} | => | {Q3=No} | 0.023 | 0.848 | 1.257 |
| [8] | {Q1=Unsatisfied, Q2=To a little extent} | => | {Q3=No} | 0.089 | 0.839 | 1.244 |
| [9] | {Q1=Very unsatisfied, Q2=To some extent} | => | {Q3=No} | 0.018 | 0.837 | 1.241 |
| [10] | {Q1=Neutral, Q2=To a little extent} | => | {Q3=No} | 0.067 | 0.836 | 1.239 |
| Nurse | | | | | | |
| [1] | {Q1=Very satisfied, Q2=To a great extent} | => | {Q3=Yes} | 0.067 | 0.925 | 2.021 |
| [2] | {Q1=Satisfied, Q2=To a great extent} | => | {Q3=Yes} | 0.050 | 0.876 | 1.915 |
| [3] | {Q1=Unsatisfied, Q2=Not at all} | => | {Q3=No} | 0.044 | 0.914 | 1.685 |
| [4] | {Q1=Neutral, Q2=Not at all} | => | {Q3=No} | 0.027 | 0.877 | 1.616 |
| [5] | {Q1=Very unsatisfied, Q2=Not at all} | => | {Q3=No} | 0.028 | 0.842 | 1.552 |
| [6] | {Q1=Unsatisfied, Q2=To a little extent} | => | {Q3=No} | 0.057 | 0.841 | 1.551 |
| [7] | {Q1=Very unsatisfied, Q2=To a little extent} | => | {Q3=No} | 0.021 | 0.830 | 1.530 |
| [8] | {Q1=Neutral, Q2=To a little extent} | => | {Q3=No} | 0.050 | 0.803 | 1.480 |
| Pharmacist | | | | | | |
| [1] | {Q1=Neutral, Q2=Not at all} | => | {Q3=No} | 0.097 | 0.935 | 1.178 |
| [2] | {Q1=Unsatisfied, Q2=Not at all} | => | {Q3=No} | 0.115 | 0.924 | 1.164 |
| [3] | {Q1=Very unsatisfied, Q2=To some extent} | => | {Q3=No} | 0.012 | 0.900 | 1.134 |
| [4] | {Q1=Very unsatisfied, Q2=Not at all} | => | {Q3=No} | 0.043 | 0.889 | 1.12 |

| **Table S4: Summaries of support, confidence, lift values for the generated association rules (continued)** | | | | | | |
| --- | --- | --- | --- | --- | --- | --- |
| Profession  [ID] | **Antecedent** {Q1: satisfaction with the current medical equipment for the management of COVID-19 outbreak, Q2: confidence in handling suspected COVID-19 patients} | **Consequent** {Q3: training for dealing  with COVID-19 patients} | | Support | Confidence | Lift |
| [5] | {Q1=Satisfied, Q2=Not at all} | => | {Q3=No} | 0.028 | 0.875 | 1.102 |
| [6] | {Q1=Unsatisfied, Q2=To a little extent} | => | {Q3=No} | 0.058 | 0.860 | 1.083 |
| [7] | {Q1=Neutral, Q2=To a little extent} | => | {Q3=No} | 0.102 | 0.854 | 1.076 |
| [8] | {Q1=Satisfied, Q2=To a little extent} | => | {Q3=No} | 0.051 | 0.844 | 1.064 |
| [9] | {Q1=Very unsatisfied, Q2=To a little extent} | => | {Q3=No} | 0.028 | 0.808 | 1.018 |
| Others | | | | | | |
| [1] | {Q1=Neutral, Q2=To a little extent} | => | {Q3=No} | 0.078 | 0.938 | 1.268 |
| [2] | {Q1=Neutral, Q2=Not at all} | => | {Q3=No} | 0.074 | 0.930 | 1.257 |
| [3] | {Q1=Unsatisfied, Q2=Not at all} | => | {Q3=No} | 0.060 | 0.903 | 1.220 |
| [4] | {Q1=Unsatisfied, Q2=To a little extent} | => | {Q3=No} | 0.067 | 0.876 | 1.185 |
| [5] | {Q1=Very unsatisfied, Q2=Not at all} | => | {Q3=No} | 0.047 | 0.865 | 1.169 |
| [6] | {Q1=Very unsatisfied, Q2=To a little extent} | => | {Q3=No} | 0.033 | 0.844 | 1.141 |

| **Table S5: Questions used to assess the knowledge of healthcare workers and response frequency** | | | |
| --- | --- | --- | --- |
| **Questions** | | **No. correct responses** | **% of total** |
| **Which of the following are symptoms of the SARS-CoV-2 infection?** | | | |
| 1 | Fever (True) | 16,521 | 95.9 |
| 2 | Cough (True) | 15,902 | 92.3 |
| 3 | Runny nose (True) | 9,499 | 55.1 |
| 4 | Sore throat (True) | 10,750 | 62.4 |
| 5 | Shortness of breath (True) | 14,420 | 83.7 |
| 6 | Joint/muscle pain (True) | 6,956 | 40.4 |
| 7 | Diarrhea (True) | 2,985 | 17.3 |
| 8 | May present with no symptoms (True) | 1,490 | 8.6 |
| 9 | Red eyes (False) | 11,323 | 65.7 |
| 10 | Rash (False) | 9,794 | 56.8 |
| **Which of the following tests should be performed for the diagnosis of SARS-CoV-2 infection?** | | | |
| 1 | Real-time polymerase chain reaction (PCR) with respiratory material (nasopharyngeal or oropharyngeal swab/ sputum/ endotracheal aspirate or bronchalveolar lavage) (True) | 14,529 | 87.1 |
| 2 | Real-time PCR with serum sample (False) | 9,759 | 58.5 |
| 3 | Chest X-ray (False) | 8,873 | 53.2 |
| 4 | Other (please specify) (False) | 15,604 | 93.6 |
| **Upon admission, which of the following should be considered to identify patients at risk of having SARS-CoV-2 infection?** | | | |
| 1 | The presence of symptoms of diarrhea (False) | 14,061 | 82.3 |
| 2 | The presence of symptoms of a respiratory infection (True) | 13,488 | 79.2 |
| 3 | History of travel to areas experiencing transmission of SARS-CoV-2 (True) | 15,003 | 88.1 |
| 4 | History of contact with possible infected patients (True) | 13,999 | 82.2 |
| **Which of the following measures should be taken to prevent transmission from known or suspected SARS-CoV-2 patients?** | | | |
| 1 | Eat boiled and cooked food (False) | 846 | 4.9 |
| 2 | Frequently clean hands by using alcohol-based hand rub or soap and water (True) | 9,075 | 52.8 |
| 3 | Put facemask on known or suspected patients (True) | 15,179 | 88.3 |
| 4 | Place known or suspected patients in adequately ventilated single rooms (True) | 12,983 | 75.5 |
| 5 | Avoid moving and transporting patients out of their area unless necessary (True) | 12,208 | 71.0 |
| 6 | Routinely clean and disinfect surfaces in contact with known or suspected patients (True) | 13,161 | 76.6 |
| 7 | All health staff members wear protective clothing (False) | 2,438 | 14.2 |

| **Table S5: Questions used to assess the preparedness of healthcare workers and response frequency (continued)** | | | |
| --- | --- | --- | --- |
| **Questions** | | **No. correct responses** | **% of total** |
| 1 | Do you feel that you keep yourself up to date on the latest information about case definitions for SARS-CoV-2 infection? (True) | 12,842 | 74.2 |
| 2 | Is there a protocol of triage and isolation in your hospital for patients with symptoms suspected of SARS-CoV-2infection? (True) | 11,893 | 74.8 |
| 3 | In your hospital, is an Airborne Infection Isolation Room (AIIR) available? (True) | 9,903 | 57.8 |
| 4 | If an Airborne Infection Isolation Room in your hospital is unavailable, do you know where to transfer a patient with suspected or confirmed SARS-CoV-2infection? (True) | 12,196 | 72.4 |
| 5 | Do you consider yourself prepared for the management of the SARS-CoV-2 outbreak? (True) | 9,820 | 57.2 |
| 6 | Do you consider your hospital prepared for the management of the SARS-CoV-2 outbreak? (True) | 9,533 | 55.9 |
| 7 | In case of contact with possible SARS-CoV-2patients, do you know how to use personal protective equipment (PPE)? (True) | 13,886 | 80.8 |
| 8 | In case of contact with confirmed SARS-CoV-2patients, do you know how to perform isolation procedures on the patients to minimize chances for exposure? (True) | 12,177 | 71.0 |
| 9 | Has your hospital established procedures for controlling visitors to known or suspected SARS-CoV-2patients? (True) | 9,066 | 53.3 |
| 10 | Do you know the precautionary measures to take when performing aerosol-generating procedures (such as tracheal intubation, non-invasive ventilation, tracheotomy, bronchoscopy, cardiopulmonary resuscitation, etc.) on SARS-CoV-2patients? (True) | 9,792 | 57.4 |
| 11 | Do you know the criteria to guide evaluation of persons under investigation (PUI) for SARS-CoV-2infection? (True) | 8,867 | 51.9 |
| 12 | Do you know where to take the report form and how to report a potential SARS-CoV-2case or exposure to facility infection control leaders and public health officials? (True) | 9,521 | 54.0 |
| 13 | Do you know who to contact in a situation where there has been an unprotected exposure to a known or suspected SARS-CoV-2patient? (True) | 12,232 | 71.4 |
| 14 | Do you know what to do if you have signs or symptoms suspected of SARS-CoV-2infection? (True) | 14,426 | 83.9 |
| 15 | Do you know who to contact (chain of command) in outbreak situations in your hospital? (True) | 13,049 | 76.1 |

**Text S1**: Detailed interpretation of Fig 4

Fig 4A shows the 10 rules (represented by circles) identified for the doctors who participated in our study. These correspond to rules (1-10) in the subsection related to doctors in the Table S4. Circles numbered 1 and 2 show how doctors who had antecedents of being "satisified/very statisifed" and who were "confident to agreat extent" {Q1: satisfied; very satisfied and Q2: To a great extent} had participated in a COVID training course {Q3: yes} with a support index (represented by the size) that ranges from 0.028 to 0.029 and a lift index ranging from 2.658 to 2.681. The remaining rules correspond with antecedents of being "neutral or unsatisfied" and "confident to some or little extent" {Q1: Neutral; unsatisfied; very unsatisfied and Q2: To some extent; To a little extent; Not at all} and not having participated in a COVID training course {Q3: No} with a support index ranging from 0.018 to 0.089 and a lift index ranging from 1.239 to 1.353. Circles (rules) 1 and 2 were generally smaller than most others, meaning that there were fewer corresponding data points, although the relationship between the antecedent and consequent is strong as reflected by darker colors than other circles.

Fig 4B shows the 8 rules generated for nurse participants and how they crosspond to rules (1-8) in the nurse subsection of Table S4. Cricles one and two show that participants who were satisified/very statisifed and confident to a great extent {Q1: satisfied; very satisfied and Q2: To a great extent} had received a COVID training course {Q3: Yes} with a support ranging from 0.050 to 0.067 and lift ranging from 1.915 to 2.021. Circles (3-8) have antecedents of not being satisfied and confident {Q1: Neutral; unsatisfied; very unsatisfied and Q2: To some extent; Not at all} and resulted in a consequent of had not received a COVID Training course {Q3: No} with a support and lift values reanging from 0.021 to 0.057 and 1.480 to 1.685, respectively. In contrast to Fig 4A, circles 1 and 2 of Fig 4B were larger and darker than the rest, meaning the antecedent and consequent relationships have a much stronger connection. Figs 4C and 4D represent the results of pharmasits and other HCWs.

**Text S2:** English version of questionnaire used in cross-sectional survey

**Questionnaire**

By completing and submitting this survey, you are indicating your consent to participate in the study. Your participation is appreciated.

**Do you agree to participate in this research project?**

⬜ Yes

⬜ No

**SECTION 1: GENERAL INFORMATION**

1. Your age in years: ..............
2. Your gender:

⬜ Male ⬜ Female ⬜ Other ⬜ Other, specify:……………….

1. The name of your hospital: .........................................
2. Your profession:

⬜ Doctor
⬜ Pharmacist
⬜ Nurse
⬜ Other, specify: ............................

1. Your working department:
   ⬜ Emergency Department

⬜ Intensive Care Unit
⬜ Outpatient Clinic
⬜ Infectious Disease Department

⬜ Respiratory Department
⬜ Other, specify: ...................

1. Your working experience as a healthcare professional (doctor, nurse, pharmacist, etc.) in years: .........

**SECTION 2: ASSESSMENT OF THE PREPAREDNESS OF HOSPITAL STAFF AGAINST THE NOVEL CORONAVIRUS 2019**

7. Have you ever experienced any outbreak (e.g., SARS, MERS, bird flu, other respiratory infection outbreak, etc.) in the past? (Select all that apply)

⬜ No
⬜ SARS
⬜ MERS
⬜ Bird flu
⬜ Other, specify: ...................

8. Has there been any confirmed human infection of 2019-nCoV in your country? (Select all that apply)

⬜ No
⬜ Yes, in my country

⬜ Yes, in my city
⬜ Yes, in my hospital

9. Please choose the sources of information from which you gained knowledge about the

SARS-CoV-2 outbreak? (Select all that apply)

⬜ Media (Newspaper, television, radio, etc.)
⬜ Social network (Facebook, Twitter, blog, etc.)
⬜ Academic Training Courses
⬜ Colleagues
⬜ Government Organization such as Ministry of Health

⬜ Other, specify: ................

10. Have you participated in any training course for dealing with the 2019-nCoV outbreak?

⬜ Yes

⬜ No

11. Which of the following are symptoms of the 2019-nCoV infection? (Select all that apply)

⬜ Fever

⬜ Cough
⬜ Runny nose
⬜ Sore throat
⬜ Shortness of breath
⬜ Joint/muscle pain
⬜ Red eyes
⬜ Rash
⬜ Diarrhea
⬜ May present with no symptoms

12. Which of the following tests should be performed for the diagnosis of SARS-CoV-2 infection? (Select all that apply)

⬜ Real-time polymerase chain reaction (PCR) with respiratory material (nasopharyngeal or oropharyngeal swab/ sputum/ endotracheal aspirate or bronchoalveolar lavage)

⬜ Real-time PCR with serum sample
⬜ Chest X-ray
⬜ Other, specify: ...............................

13. Do you keep yourself up to date on the latest information about case definitions for SARS-CoV-2infection?

⬜ Yes

⬜ No
14. Is there a protocol of triage and isolation in your hospital for patients with symptoms suspected of SARS-CoV-2infection?

⬜ Yes

⬜ No

⬜ I don’t know

15. Upon admission, which of the following should be considered to identify patients at risk of having SARS-CoV-2infection? (Select all that apply)

⬜ The presence of symptoms of diarrhea
⬜ The presence of symptoms of a respiratory infection
⬜ History of travel to areas experiencing transmission of 2019-nCoV

⬜ History of contact with possible infected patients

16. In your hospital, is an Airborne Infection Isolation Room (AIIR) available?

⬜ Yes

⬜ No

⬜ I don’t know
17. If an Airborne Infection Isolation Room in your hospital is unavailable, do you know where to transfer a patient with suspected or confirmed SARS-CoV-2infection?

⬜ Yes

⬜ No
18. Do you consider yourself prepared for the management of the SARS-CoV-2outbreak?

⬜ Yes

⬜ No
19. Do you consider your hospital prepared for the management of the SARS-CoV-2outbreak?

⬜ Yes

⬜ No
20. Please rate how satisfied you are with the current medical equipment in your hospital for the

management of the SARS-CoV-2outbreak:

⬜ Very unsatisfied

⬜ Unsatisfied
⬜ Neutral

⬜ Satisfied

⬜ Very satisfied

21. In case of contact with possible SARS-CoV-2patients, do you know how to use personal protective equipment (PPE)?

⬜ Yes

⬜ No
22. In case of contact with confirmed SARS-CoV-2patients, do you know how to perform isolation procedures on the patients to minimize chances for exposure?

⬜ Yes

⬜ No
23. To what extent are you confident in handling suspected SARS-CoV-2patients?

⬜ No confidence at all
⬜ To a little extent

⬜ To some extent
⬜ To a considerable extent
⬜ To a great extent

24. Has your hospital established procedures for controlling visitors to known or suspected SARS-CoV-2 patients?

⬜ Yes
⬜ No
⬜ I don’t know

25. Which of the following measures should be taken to prevent transmission from known or suspected SARS-CoV-2patients? (Select all that apply)

⬜ Frequently clean hands by using alcohol-based hand rub or soap and water

⬜ Eat boiled and cooked food
⬜ Put facemask on known or suspected patients
⬜ Place known or suspected patients in adequately ventilated single rooms

⬜ All health staff members wear protective clothing

⬜ Avoid moving and transporting patients out of their area unless necessary

⬜ Routinely clean and disinfect surfaces in contact with known or suspected patients

26. Do you know the precautionary measures to take when performing aerosol-generating procedures (such as tracheal intubation, non-invasive ventilation, tracheotomy, bronchoscopy, cardiopulmonary resuscitation, etc.) on SARS-CoV-2patients?

⬜ Yes

⬜ No
27. Do you know the criteria to guide evaluation of persons under investigation (PUI) for SARS-CoV-2 infection?

⬜ Yes

⬜ No
28. Do you know where to take the report form and how to report a potential SARS-CoV-2case or exposure to facility infection control leaders and public health officials?

⬜ Yes

⬜ No
29. Do you know who to contact in a situation where there has been an unprotected exposure to a known or suspected SARS-CoV-2patient?

⬜ Yes

⬜ No
30. Do you know what to do if you have signs or symptoms suspected of SARS-CoV-2infection?

⬜ Yes

⬜ No
31. Do you know who to contact (chain of command) in outbreak situations in your hospital?

⬜ Yes

⬜ No

32. What is your suggestion for improving the preparedness of health staff against SARS-CoV-2 in your hospital?

................................................................................................................................................................................................................................................................................................................................................................

THANK YOU FOR TAKING THE SURVEY

**Text S3:** Contributors of TMGH-Global COVID-19 Collaborative

**Study guarantors:** Nguyen Tien Huy (Email: tienhuy@nagasaki-u.ac.jp; School of Tropical Medicine and Global Health, Nagasaki University, Japan) and R Matthew Chico (Email: Matthew.Chico@lshtm.ac.uk; London School of Hygiene & Tropical Medicine, UK). Nguyen Tien Huy is the lead of the TMGH-Global COVID-19 Collaborative.

**Project Managers:** Vuong Thanh Huan (Pham Ngoc Thach University of Medicine, Vietnam), Hosam Waleed Shaikhkhalil (Islamic University of Gaza, Palestine), Vuong Ngoc Thao Uyen (International University - Vietnam National University, Ho Chi Minh city, Vietnam), Ahmad Taysir Atieh Qarawi (Lower Westchester Medical Associates, P.C., USA), Shamael Thabit Mohammed Alhady (University of Gezira, Sudan), Nguyen Lam Vuong (University of Medicine and Pharmacy at Ho Chi Minh City, Vietnam), Le Van Truong (Traditional Medicine Hospital of Ministry of Public Security, Vietnam), Mai Ngoc Luu (University of Medicine and Pharmacy at Ho Chi Minh City, Vietnam), Shyam Prakash Dumre (Institute of Tropical Medicine, Nagasaki University, Japan), Atsuko Imoto (School of Tropical Medicine and Global Health, Nagasaki University, Japan), Peter N Lee (P.N.Lee Statistics and Computing Ltd, UK), Dao Ngoc Hien Tam (Asia Shine Trading & Service CO, LTD., Vietnam), Sze Jia Ng (Universiti Sains Malaysia, Malaysia), Mohammad Rashidul Hashan (Government of the People's Republic of Bangladesh - Ministry of Health and Family Welfare, Bangladesh), Mitsuaki Matsui (School of Tropical Medicine and Global Health, Nagasaki University, Japan), Nguyen Tran Minh Duc (University of Medicine and Pharmacy at Ho Chi Minh City, Vietnam), Sedighe Karimzadeh (Sabzevar University of Medical Sciences, Sabzevar, Iran), Nut Koonrungsesomboon (Chiang Mai University, Thailand), Chris Smith (School of Tropical Medicine and Global Health, Nagasaki University, Japan; London School of Hygiene & Tropical Medicine, UK), Sharon E Cox (School of Tropical Medicine and Global Health, Nagasaki University, Japan; London School of Hygiene & Tropical Medicine, UK), Kazuhiko Moji (School of Tropical Medicine and Global Health, Nagasaki University, Japan), Kenji Hirayama (Institute of Tropical Medicine, Nagasaki University, Japan), Le Khac Linh (VinUniversity, Vietnam), Kirellos Said Abbas (Alexandria University, Egypt), Tran Nu Thuy Dung (University of Medicine and Pharmacy at Ho Chi Minh City, Vietnam), Tareq Mohammed Ali AL-Ahdal (Jordan University of Science and Technology, Jordan), Emmanuel Oluwadare Balogun (Ahmadu Bello University, Nigeria), Nguyen The Duy (University Hospital Giessen and Marburg, Germany), Mennatullah Mohamed Eltaras (Al-Azhar University, Egypt), Trang Huynh (University of Medicine and Pharmacy at Ho Chi Minh City, Vietnam), Nguyen Thi Linh Hue (Nguyen Trai Hospital, Vietnam), Bui Diem Khue (University of Medicine and Pharmacy at Ho Chi Minh City, Vietnam), Abdelrahman Gad (Ain Shams University, Egypt), Gehad Mohamed Tawfik (Ain Shams University, Egypt), Kazumi Kubota (Yokohama City University, Japan), Hoang-Minh NGUYEN (University of Medicine and Pharmacy at Ho Chi Minh City, Vietnam), Dmytro Pavlenko (Bogomolets National Medical University, Kyiv, Ukraine), Vu Thi Thu Trang (National Hospital of Traditional Medicine, Vietnam), Le Thuong Vu (University of Medicine and Pharmacy at Ho Chi Minh city, Vietnam), Hai-Yen Tran (International University - Vietnam National University, Ho Chi Minh city, Vietnam), Nguyen Thi Yen-Xuan (Cambridge University Hospital Foundation Trust, UK), Luong Thi Trang (Danang Oncology Hospital, Vietnam), Vinh Dong (American University of the Caribbean, Sint Maarten), Akash Sharma (University College of Medical Sciences & Guru Teg Bahadur Hospital, Dilshad Garden, Delhi, India), Vu Quoc Dat (Hanoi Medical University, Vietnam), Mohammed Soliman (Zagazig University, Egypt), Jeza Muhamad Abdul Aziz (University of Human Development, Sulaimani, Iraq), Jaffer Shah (Drexel University College of Medicine, Pennsylvania, USA), Pham Dinh Long Hung (University of Medicine and Pharmacy at Ho Chi Minh City, Vietnam), Yap Siang Jee (Universiti Sains Malaysia, Malaysia), Dang Thuy Ha Phuong (Pham Ngoc Thach University of Medicine, Vietnam), Tran Thuy Huong Quynh (Kansai Medical University, Osaka, Japan), Hoang Thi Nam Giang (The University of Danang, Vietnam), Vy Thi Nhat Huynh (University of Debrecen, Hungary), Nguyen Anh Thi (Toulouse III Paul Sabatier University, France), Nacir Dhouibi (University of Tunis El Manar, Tunisia), Truc Phan (Vinmec International Hospital, Vietnam), Duru Vincent C (Nnamdi Azikiwe University, Nigeria), Nguyen Hai Nam (Kyoto University, Japan), Sherief Ghozy (Mansoura University, Mansoura, Egypt).

**National leads:**

**Afghanistan**: Sayed Hamid Mousavi (Afghanistan National Charity Organization for Special Disease), Shafi Ullah Zahid (Jamhuriat Hospital), Kalimullah Wardak (Wazir Akbar Khan Hospital);

**Bangladesh**: Shyam Prakash Dumre (Nagasaki University), Farhana Nusrat (Sylhet MAG Osmani Medical College);

**Brazil**: Zainab Gandhi (C.U. Shah Medical College);

**Cameroon**: Joel Noutakdie Tochie (University of Yaounde 1);

**Canada**: Michael G. Blennerhassett (Queen’s University);

**China**: Yap Siang Jee (Universiti Sains Malaysia), Kefang Wang (Shandong University), Hongcan Shi (Yangzhou University);

**Chile:** Dmytro Pavlenko (Bogomolets National Medical University, Ukraine);

**Ecuador**: Estefanía Ochoa-Toasa (Laboratorio Clínico, Pontificia Universidad Católica del Ecuador, Laboratorio de Especialidades Medicas Ochoa&Ochoa), Shyam Prakash Dumre (Nagasaki University);

**Egypt**: Gehad Mohamed Tawfik (Ain Shams University), Kirellos Said Abbas (Alexandria University), Mostafa Elsayed Elsayed Hewalla (Alexandria University), Fatma A. Monib (Assiut University), Mahmoud Ali Alfadaly (Al-Azhar University-Damietta), Mona Hanafy Mahmoud (Ain shams university), Ezz eldeen Atef Derballa (Fayoum University);

**Ethiopia**: Zelalem Desalegn (Addis Ababa University), Tewodros Tesfa (Haramaya University);

**Hong Kong**: Rex Pui Kin Lam (The University of Hong Kong);

**India**: Akash Sharma (Guru Teg Bahadur Hospital), Shyam Prakash Dumre (Nagasaki University), Zahiruddin Quazi Syed (Datta Meghe Institute of Medical Sciences);

**Indonesia**: Harapan Harapan (Universitas Syiah Kuala), Ahmad Taysir Atieh Qarawi (Lower Westchester Medical Associates, P.C.);

**Iran**: Ali Khanmohamadi Hezave (Arak University of Medical Sciences);

**Iraq**: Mohammed Baqer Al-Jubouri (University of Baghdad), Selman Hussain Faris (University of Kerbala), Jeza Muhamad Abdul Aziz (University of Human Development, Sulaimani), Sadeq Al-Fayyadh (University of Baghdad), Salwa Ghazi Turki (University of Baghdad), Sabah Abdullah Jaafar (University of Al-Muthana), Iman Hussein Alwan (University of Baghdad);

**Italy**: Fabio Porru (Erasmus Medical Center, Rotterdam, The Netherlands), Stefano Greco (University of L'Aquila, Italy);

**Japan**: Kazumi Kubota (Yokohama City University), Atsuko Imoto (Nagasaki University), Nahoko Harada (University of Miyazaki), Anna Kubota (Keio University), Yoshiaki Iwashita (Shimane University Hospital), Takeshi Mizusawa (Niigata University), Emmanuel Oluwadare Balogun (Ahmadu Bello University, Nigeria);

**Jordan**: Tareq Mohammed Ali AL-Ahdal (Jordan University of Science and Technology), Mallak Alomoush (Hashmite University), Tareq Osaili (Jordan University of Science and Technology);

**Libya**: Marwa Saed Ali Emhamed (University of Tripoli);

**Malaysia**: Sze Jia Ng (Hospital Enche' Besar Hajjah Khalsom) , Ilham Ameera Ismail (Universiti Teknologi Mara), Kek Heng Chua (University of Malaya), Tengku Zetty Maztura Tengku Jamaluddin (Universiti Putra Malaysia);

**Mexico:** Dmytro Pavlenko (Bogomolets National Medical University, Ukraine), Brianda del Pilar Gómez Olvera (Autonomous University of Mexico State);

**Morocco:** Oumaima Outani (Faculty of Medicine and Pharmacy of Rabat);

**Nepal**: Shyam Prakash Dumre (Nagasaki University), Kamal Ranabhat (Tribhuvan University), Renu Bhandari Dumre (Nagasaki University);

**Nigeria**: Farouq Muhammad Dayyab (Infectious Diseases Hospital), Ahmad Abdulmajid Yakubu (Abubakar Tafawa Balewa University), Duru Vincent C (Nnamdi Azikiwe University), Deborah Tolulope Esan (Afe Babalola University), Pembi Emmanuel (State Ministry of Health, Adamawa State), Ahmadu Baba Usman (Federal Medical Centre Yola);

**Pakistan**: Ramesh Kumar (Health Services Academy Ministry of Health);

**Palestine**: Hosam Waleed Shaikhkhalil (Islamic University of Gaza), Ahmad Taysir Atieh Qarawi (Lower Westchester Medical Associates, P.C., USA), Emad I H Shaqoura (Islamic University of Gaza), Taj El-deen Shaat (Islamic University of Gaza), Loay Hidar Hamad (Islamic University of Gaza), Abdullah Rasmi Awwad (Islamic University of Gaza), Ahmad Samara (An-Najah National University);

**Philippines**: John Robert Carabeo Medina (University of the Philippines Manila), Allan Laurio Hilario (University of the Philippines Manila), Jose Ma. Moncada Angeles (University of the Philippines Manila), Fresthel Monica Marqueses Climacosa (University of the Philippines Manila), Van Jerwin Purificacion Mercado (University of the Philippines Manila);

**Russia:** Dmytro Pavlenko (Bogomolets National Medical University, Ukraine);

**Serbia**: Srdjan Stefanovic (University of Kragujevac);

**South Africa**: Indiran Govender (University of Pretoria);

**Spain**: Raigam Jafet Martinez-Portilla (Institut Clínic de Ginecologia, Obstetricia i Neonatologia);

**Sri Lanka**: N.D.B. Ehelepola (Teaching Hospital-Peradeniya), Rohitha Muthugala (Regional Virology Laboratory, Kandy);

**Sudan**: Anmar Homeida (Federal Ministry of Health), Shamael Thabit Mohammed Alhady (University of Gezira), Omer Ahmed (Federal Ministry of Health), Asma Yahya Ismail (Sudan International University);

**Syria**: Mosa Shibani (Syrian Private University), Basel Kouz (Damascus University);

**Taiwan**: Wen-Chien Ko (National Cheng Kung University Hospital);

**Thailand**: Surapon Nochaiwong (Chiang Mai University), Nut Koonrungsesomboon (Chiang Mai University), Shyam Prakash Dumre (Nagasaki University);

**Tunisia**: Nesrine Ben Hadj Dahman (University of Tunis El Manar);

**United Arab Emarites:** Tareq Osaili (Jordan University of Science and Technology);

**Ukraine:** Dmytro Pavlenko (Bogomolets National Medical University, Ukraine), Tetiana Pavlenko (Oleksandriia Central City Hospital), Roman Pavlenko (Bogomolets National Medical University);

**United States of America**: George L. Anesi (University of Pennsylvania);

**Vietnam**: Truc Phan (Vinmec International Hospital), Tran Thuy Huong Quynh (Kansai Medical University, Osaka, Japan), Dang Thuy Ha Phuong (Pham Ngoc Thach University of Medicine), Luong Thi Trang (Danang Oncology Hospital), Pham Ba Tuyen (Traditional Medicine Hospital of Ministry of Public Security, Vietnam);

**Zambia**: Duncan Chanda (University Teaching Hospital);

**Local collaborators:**

**Afghanistan**: Hamed Shenwari (Jamhuriat Hospital); Sayed Maseehullah Hashimi (Rabia-e-Balkhi Hospital); Farwa Afzaly (Blossom Hospital); Abdul Wahid Noor (Ali Abad Hospital); Nasrat Alizai (Maiwand Hospital);

**Albania**: Irida Dajti, Jola Kërpaçi, Enxhi Vrapi (University Hospital of Obstetrics – Gynecology " Koço Gliozheni");

**Algeria**: Yassamine Ouerdane, Fella Ouerdane, Mohamed ElKhalil Bouaich, Imane Bakhtaoui (University Blida);

**Australia**: Dominic E Dwyer, Janette Taylor (Westmead Hospital);

**Bangladesh**: Orindom Shing Pulock, Susmita Dey Pinky, Tanjimul Hye Rafi, Senjuti Deepanwita (Chittagong Medical College); Alvee Ahsan, Raisa Nawal Mahboob (Mymensingh Medical College); Abdur Rafi, Ahsan Habib, Mohammad Fahim Faisal (Rajshahi Medical College); Soumik Kha Sagar, Farhana Rahman Luba, Saiful Islam Sourav, Sajibur Rahman (Sylhet MAG Osmani Medical College);

**Brazil**: Zainab Gandhi (C.U. Shah Medical College); Lirane Elize Defante Ferreto (Western Paraná State University), Moacir Antonio de Pauli Junior, Mirian Carla Bortolamedi da Silva (Hospital Regional do Sudoeste do Paraná); Guilherme Welter Wendt (Western Paraná State University);

**Cameroon**: Tebit Emmanuel KWENTI, Kouamo Gisele Wendy KAM (University of Buea); Nelson Njinyam (Banyo District Hospital); Elvins Tsaku (Centre Medical d'Arrondissement de Ngaoundal);

**Canada**: Nahal Farhani, Azar Bayat, Faith Tabije, Samreen Rizwan, Loreanne Kathleen Baino Manalac, Navreet Kaur Ubhi (Queen’s University);

**Chile**: José Tomás Ordóñez Aburto (University of La Frontera);

**China**: Jing Mou, Shouwei Zhang (The People’s Hospital of Rizhao); Cuiping Xu, Jianhong Qiao, Xingfeng Lin, Feifei Chen, Xiaorong Luan, Min zhang (Shandong University); Xiuli Wang, Li Chen (Shandong Provincial Third hospital); Xuejuan Cheng, Cuihua Song (The People's Hospital of Zouping City); Ling Guo (Yidu central hospital of Weifang); MD Ariful Haque, Xiong Ying (Yan'an Affiliated Hospital of Kunming Medical University); Yongxiang Wang (Subei People's Hospital); Jihong Lu (Yangzhou Hospital of Traditional Chinese Medicine); Kaizheng Gong (Yangzhou First People's Hospital); Ying Zheng, Hongjuan Liu, Wenji Li (Yangzhou University); Wong Tin Wui (Universiti Teknologi MARA, Malaysia);

**Ecuador**: Gabriela Anasi Castillo, Santiago Ruales, María Marcela Bovera (Hospital de los Valles); Danny Patricio Flores Almeida, Denisse Miroslava Costales Quiroz (Hospital General Docente de Calderón); Ana Paola Capelo Rodriguez, Micaela Alejandra Navarrete Rengel, Oscar David Salazar Correa (Clínica Infes); Lucia Jeannete Zurita Salinas, Juan José Romero Carvajal, Cinthya Verónica Delgado Arteaga (Hospital Vozandes Quito);

**Egypt**: Rania Abdel Maguid, Hoda Aly Mohamed Omran, Ahmed Abdulgalil Yousif Mohammed, Atef Khairy Sharaf, Adel Reda Mustafa Hebashy, Reda Maher Moussa, Toka Aziz Fayze El-Ramly, Asmaa Reda Ahmed Ahmed Youssef, Mohamed Ibrahim Abdo Ibrahim, Chiristine Samuel Rezq, Mirna Ragy Shoukry, Mostafa Shehata Qatora, Mohammed Jawad Abuhassira, Mina Ragy Shoukry, Mostafa Mohamed Bastawesy, Ahmed ElSayed Bakry Mohamed Abdeltawab, Ayman Hafez Y, Mohamed A.Mekkawy (Alexandria University); Sara Mohammed Hashem, Amira Mohammed Mahmoud, Esraa G. Sayed, Mahmoud M.Saad, Abdelrahman G. Ramadan, Ahmed Mohamed Rashad, Aliaa Effat Said, Shehab Fathy Ahmed, Ziad Hassan Hamed, Mohamed Ashraf Salah, Mohamed Mahmoud Abdelkarem, Mahmoud Usama Fawzy, Rana Mahmoud Farghali, Nashwa Rafaat Foaud, Mariam Albatoul Nageh (Assiut University); Ahmed Hafez Saif Allam, Abdelrahman Amin Mahmoud Abdallah, Ahmed Gameel Nassef Abdallatif (Menoufia University); Mohamed Eid Alsadek, Mohammad Saad Isa, Ahmed Yousof, Alaa Saad Isa, Fathiya El-Raey, Abdallah Moustafa Ghanem, Atef Wahdan El-Rifai, Mennatullah Mohamed Eltaras, Ahmed Elsayad Salama, Mahmoud Abdulmonem Abulnaga, Nourhan Mohamed Hamdy, Aya Ali Abuzeid, Sarah Abd Elaziz Khader, Mai Alaa Eldin Temraz Elsebaie, Bassant Mohsen Taher Ragab, Youssef ElSaid Mohamed Shady, Abdallah Ashraf Mahmoud Ali, Abdelrahman Ashraf Mahmoud Ali, Dina Emad Said, Hawwa Abdullah Salih Albishari, Najma Abdelhady Mansour Mohammed, Eslam Mohamed Abd Elsalam (Ain Shams University); Noha Ali Abdelsamiee Ali (Al-Azhar University); Mohammed Salah Desokey, Abdelrahman Ahmad Elsamman, Ahmed Zakaria Khaleel, Mohamed Zaki Ali Eldahshory, Mohamed Moustafa Hussein (Aswan University Hospital); Sara S. Elsheikh, Dalia A. Deeb, Mohamed Refaat Badr (Zagazig University); Mohamed Fouad Ibrahim Abdrabo, Mohammed Maher Hadhoud (Tanta University); Gehad Taha Abdelwadoud, Nada Khaled Abdelsattar, Aya Ghiath Alrawi, Marwa Gamal Mustafa, Menna-Allah Sayed kamal, Hanaa Abdelmonem Hussien, Manar Mohammed Hosny, Taha Abdelsalam Ashraf Taha Abdelsalam, Alshaimaa Galal Mohamed, Hajar Ahmed Arafa, Mohamed Mahmoud Abd-ElGawad, Omnia Mohamed Abd Elsalam, Mahmoud Ahmed Farag, Radwa Hamdy Allam, Esraa Mostafa Kamal, Asmaa Khaled Mostafa, Mostafa Ramadan Mohamed, Habiba Safwat Ewais, Abdullah Ahmed Ahmed, Ahmed Taher Masoud, Mohamed Abdelmonem, Fatma Mohamed Sayed Manar Hamdy Mohammed, Ahmed Fares Ghannam (Fayoum University);

**Ethiopia**: Yirgu Gebrehiwot, Wondwossen Amogne (Tikur Anbessa Specialized Hospital); Tamrat Abebe (Addis Ababa University); Alem Abrha (Yekatit-12 Hospital Medical College); Mesay Arkew (Haramaya University); Daniel Gebretsadik (Wollo University); Chilot Yefredew (Dil-chora Referral Hospital);

**France**: Nguyen Duc, Malvy Denis, Perreau Pauline (University Hospital of Bordeaux); Constantin Jean-Michel (Sorbonne University);

**Germany**: Andreas Hecker, Matthias Hecker, Natascha Sommer (University Hospital of Giessen);

**Greece**: Dimitrios K. Manatakis, Panagiotis Vamvakas, Eleni Milisi (Athens Naval and Veterans Hospital);

**Guatemala**: Angel Alfonso Velarde Lopez, Ingrid Fabiola Castillo, Vicky De Falla (Liga Nacional Contra El Cáncer-INCAN);

**Hong Kong**: Grace Sin Man LAM (Pamela Youde Nethersole Eastern Hospital); Patrick Siu Chung LEUNG, Pauline Yeung NG, Wai Ching Simon SIN (The University of Hong Kong);

**India**: Subramaniam R, Suneesh Kuruvilla (Indira Gandhi Institute of Dental Sciences, Kothamangalam); Supram Hosuru Subramanya (Manipal College of Medical Sciences, Nepal); Ambica Rangaiah, Shashiraja Padukone (Bangalore Medical College and Research Institute); Suhrud Panchawagh, Shreepad Bhat (Smt. Kashibai Navale Medical College and General Hospital, Pune); Akshay Raut (R.C.S.M. Government Medical College, India); Ketty E Arce, Gerardo Alvarez-Uria (Rural Development Trust Hospital Bathalapalli); Sudhindra Baliga (Sharad Pawar Dental College); Chandrashekar Mahakalkar (Jawaharlal Nehru Medical College, Wardha);

**Indonesia**: Prattama Santoso Utomo (Universitas Gadjah Mada); Nurfanida Librianty (Universitas Indonesia); Ikram Ikram (Dr H Yuliddin Away Hospital); Nyoman Ananda Putri Prashanti (Bangli Hospital); Tri Novita Wulan Sari (Sungai Dareh Hospital);

**Iran**: Mehdi Harorani, Saeed Amini (Arak University of Medical Sciences); Behnam Masmouei (Shiraz University of Medical Sciences);

**Iraq**: Safad Riyadh Isam, Asmahan Adnan Abbas (University of Baghdad); Hayder Alaa Ahmed (Al-Nahrain University); Haneen Alaa Ahmed (Central Hospital for Pediatrics); Rangin Muhamad Hussein, Dashne Jalal Hama (Baxshin Hospital); Trifa Abdalla Mahmood, Darya Saeed Abdulateef, Heshu Sulaiman Rahman, Hemn Hassan Othman (University of Sulaimani); Dana Taib Gharib (Ministry of Health/ Directory of Sulaimani/ KCGH); Kawa Faraj Saadun (Respiratory and Chest Diseases Center, Asthma and Allergy Center); Staar Mohammed Qader (Tikrit University); Ahmed Saleh Hilal (North Oil Company- K1 Hospital); Hassan Abdullah Athbi, Ali Kareem Khudhair, Khamees Bandar Obaid, Ali Jabbar Abd Al-Husayn, Maytham Fawzy Salman, Haqi Ismael Mansoor, Zeki Sabah Musihb, Sajida Saadoon Oleiwi, Fatma Makee Mahmood (University of Kerbala); Ismael Hasan Jawad (Al Hilla Teaching Hospital); Raad Farhan Jabl (Ibn Al-Nafees Teaching Hospital);

**Italy**: Simone Piva, Ciro Paolillo (Spedali civili di Brescia); Sandro Mancinelli, Antonio Vinci, Fabio Ingravalle (University of Rome "Tor Vergata"); Maria Michela Gianino, Daniela Acquadro Maran (University of Torino), Riccardo Papalia (San Martino Hospital, Genova), Flavio Boraso (Azienda Sanitaria Locale Torino 3); Francesco Rosiello (University of Rome "La Sapienza"); Rosa Costantino, Gianluca Raffaele (Azienda Ospedaliera "Pugliese Ciaccio"); Aida Bianco, Davide Pepe, Francesca Licata, Silvia Mazzea ("Magna Græaecia" University of Catanzaro); Caterina De Filippo (Azienda Ospedaliera Universitaria "Mater Domini"); Davide Brunelli, Matteo Verzè, Fabrizio Nicolis (IRCCS Sacro Cuore Don Calabria Hospital); Diego Alberto Ramaroli, Stefano Tardivo (University of Verona); Angela Currà, Raffaele Bava (Ospedale Giulio Jazzolino); Antonella Rodinò, Matteo Galletta (Azienda Ospedaliera "Bianchi-Melacrino-Morelli”); Stefano Formentini, Mario Capasso (Ospedale Ca’ Foncello di Treviso);

**Korea:** Won Suk Choi (Korea University College of Medicine);

**Japan**: Yoshiaki Iwashita (Shimane University Hospital); Asami Ito (Mie University Hospital); Shinnosuke Morimoto (Kinan Hospital); Shinji Akitomi (National Defense Medical College); Rintaro Sawa (The Japan Medical Association Research Institute); Kaku Tamura (Japan Self-Defense Forces Central Hospital); Sachiyo Nagi, Tomohiko Sugishita, Daisuke Tokita, Kazunari Tanabe (Tokyo Women's Medical University); Eri Tanaka, Chie Minamide, Kumiko Maeda (Hayama Heart Center); Hiromi Munakata, Makoto Hibino, Rie Suzuki (Shonan Fujisawa Tokushukai Hospital); Nobuaki Shinozaki, Morihiko Sato, Nobuyo Watanabe (Shonan Kamakura General Hospital); Yoshito Kadoya (Kyotango City Yasaka Hospital); Fumihiro Hayakawa (Shinshiro Municipal Hospital); Fumitoshi Yoshimine, Yoshihisa Hori, Youko Sugai (Niigata Prefectural Tokamachi Hospital); Yukiyoshi Kon, Sho Shimazu (Niigata Prefectural Sakamachi Hospital); Hayato Tsuruma, Atsushi Uchiyama (Sado Municipal Ryotsu hospital); Kyuma Ota (Niigata Prefectural Kakizaki Hospital); Kenji Takehara, Isao Miyairi (National Center for Child Health and Development); Shingo Kawaminami, Keisuke Kawahito (Tokushima Prefectural Kaifu hospital); Shusaku Mizukami (Nagasaki University); Joji Kayano, Kazuhiro Tanaka (Nagasaki Yuuai Hospital);

**Jordan**: Reem Hazza Ahmed Yahya Al-Jawlahi, Yousef Saleh Khader, Suleiman M. Momany, Faris Jamal ALAbed Abu Za'nouneh, Qusai Mohammad AlQudah, Ayham Mohammad Al Momani, Anas Abdel-Rauof Al-Nabulsi (Jordan University of Science and Technology); Fedaa Taha AlGhammaz (Irbid Specialty Hospital); Mohannad Diraneih (Amman Arab University); Khaled Ahamad Hasanein (Al-Essra Hospital); Jehad Sahli, Dania Alaawneh (The Hashemite University); Mo’men Ali Sisan (King Hussein Cancer Centre);

**Lebanon**: Ghassan Al-Awar, Georges Minassian, Rita Ayoub, Joelle Hassanieh (American University of Beirut);

**Libya**: Aliyah Mustafa Alshareef, Malak Masoud Ahmed Alduwayb, Safa Abdalhakem Elrais, Mohamed Omar M. Alhabbasi, Faeq Alhadi Ahmed, Adnan Alhadi Salah Ben-Abdallah, Marwa Mohammed Morgom, Muaad Alhussein Etturki (University of Tripoli); Mohammed Khayri Aboubeirah (Elmegib University);

**Malaysia**: Khasnur Abd Malek, Nur Fazlinda Md Shah (Universiti Teknologi MARA); Suat Moi Puah, Vijayan Munusamy, Ong Hang Cheng, Suvarnesh Tarumma, Siti Zuhairah Mohamad Razali, Mohd Afiq Mohd Nor, Sasheela Ponnampalavanar (University of Malaya); Ping Chin Lee, Jennifer Kui Ling Chee, Alvin Oliver Payus (Universiti Malaysia Sabah); Ping Yein Lee, Siti Zulaikha Zakariah, Rosni Ibrahim, Niazlin Mohd Taib, Syafinaz Amin-Nordin, Muhammad Mohd Isa, Aditya Adawiah Darwis, Fauziah Adnan (Universiti Putra Malaysia); Aishah Hani Azil, Umi Kalsom Ali, Azlin Mohd Yasin, Sharifah Azura Salleh (Universiti Kebangsaan Malaysia);

**Mexico:** Brianda del Pilar Gómez Olvera (Autonomous University of Mexico State);

**Morocco**: Zaineb Benslimane, Lina Boualila, Ihsane Skitioui, Belyamani Lahcen (Faculty of Medicine and Pharmacy of Rabat); Ghassane El Adib, Hamza Belakbyer (Faculty of Medicine and Pharmacy of Marrakech); Aymane Outani (Abokrat Health Institute); Ahmed Ayad (Faculty of Medicine Kasr Alainy);

**Nepal**: Kalyan Sapkota, Bhojraj Adhikari, Bijaya Dhakal (Bharatpur Hospital); Basu Dev Pandey, Anup Bastola, Sher Bahadur Pun, Shrawan Kumar Mandal (Sukraraj Tropical and Infectious Diseases Hospital); Pradeep Vaidya, Pradip Gyanwali, Amod Kumar Poudyal, Komal Raj Rijal, Prakash Ghimire (Tribhuvan University); Sujan Babu Marahatta, Durga Khadka Mishra (Manmohan Memorial Institute of Health Sciences); Dhiraj Acharya (The University of Chicago); Pukar Ghimire, Madhav Ghimire (College of Medical Sciences Teaching Hospital); Deelip Raj Neupane, Samip Thapaliya (Parkland Hospital); Roshan Chhetri, Deebya Raj Mishra, Shailesh Mani Pokharel (BP Koirala Institute of Health Sciences); Bidesh Bista, Samir Neupane, Bishesh Sharma Poudyal (Civil Hospital); Kishor Pandey (Nepal Academy of Science and Technoogy); Shishir Gokhale, Deependra Hamal, Rajani Shrestha (Manipal College of Medical Sciences); Kedar Prasad Ceintury, Kishor Bhandari, Achyut Raj Karki (Bir Hospital); Bishwanath Acharya (Thammasat University); Pardip Kumar Oli, Durga Laxmi Shrestha (Bheri Zonal Hospital); Mahendra Prasad Shrestha, Bibek Kumar Lal, Dipendra Raman Singh (Ministry of Health and Population); Meghnath Dhimal (Nepal Health Research Council);

**Netherlands**: Yvette Loeffen (Pediatrician ID Specialist);

**New** **Zealand**: Ibrahim S. Al-Buasidi (Christchurch School of Medicine, University of Otago); Sarah Metcalf (Christchurch Hospital);

**Nigeria**: Farouq Muhammad Dayyab (Infectious Disease Hospital); Garba Iliyasu (Aminu Kano Teaching Hospital); Abdulrazaq Garba Habib (Bayero University); Bashir Garba Ahmad, Faiza Sadauki Kibiya, Sani Umar (Muhammad Abdullahi Wase Teaching Hospital); Awwal Musa Borodo, Sulaiman Ibrahim Sulaiman, Amira Muhammad Awwal (Murtala Muhammad Specialist Hospital); Yunusa Sanusi (Hasiya Bayero Paediatric Hospital); Abdurrahman A Sadiq, Zuwaira Hassan, Ibrahim Mahmood Maigari (Abubakar Tafawa Balewa University Teaching Hospital); Ahmad Abdulmajid Yakubu, Hassan Shuaibu Musa, Yusuf Abdu Misau (Abubakar Tafawa Balewa University Clinic); Aisha Abdulmalik Abdullahi, Habiba Ismail, Adamu Muhammad (State Specialist Hospital Bauchi); Zulfa'u Abubakar Isah, Sumayya Muhammed (Primary Healthcare Center Kofar Ran Bauchi); Lukman Muhammad, Abdullahi Sani Gadama (Bayero University); Bako Abubakar Muhammad (Infectious Disease Hospital Bayara Bauchi); Muhammad Mustapha Mubarak, Murtala Abubakar, Muhammad Abdullahi Umar (General Hospital Gamawa Bauchi State); Robinson Yusuf, Adamu Lawiza Haruna, Khalid Abubakar Isah (Private Healthcare provider and Women and children Hospital); Dr Muhammad Jamilu Dauda, Sulaiman Aminu Tukur (Almanzoor Hospital Bauchi); Babatunde Adewale, Olusola Ajibaye, Adewale Ojogbede (Nigerian Institute of Medical Research); Bolanle Olarewaju Awoyomi (Federal Medical Centre); Adejuwon Adewale Adeneye (Lagos State University College of Medicine); Olawunmi Victoria Ajibaye (Lagos University Teaching Hospital); Oluwatoyin Ganiyu Olawale, Elon Isaac, Mohammed M Manga (Federal Teaching hospital, Gombe); Deborah Tolulope Esan, Tunrayo Oluwadare, Funmilayo Stella Oluwafemi (Afe Babalola University); Theophilus Olaide Esan, Segun Matthew Agboola (Federal Teaching Hospital, Ido-Ekiti); Lawal Ismail Akinlade, O. A Osinupebi, Osisanwo Deborah (Olabisi Onabanjo University Teaching Hospital); Oduyebo Oyinlola O, Olise Kingsley Ejechi, Adewole Monisade Francisca (Lagos University Teaching Hospital); Ahmadu Baba Usman (Federal Medical Centre (F.M.C) Yola); Pembi Emmanuel, Abdulmalik Yusufu Kadabiyu G. Jones, Owoseni Olufunke Abosede, Francis Ashesla Mercy, Okon Nsikak Joseph (State Ministry of Health, Adamawa State); Philip ThankGod Duru (Specialist Hospital Yola); Bulus Nuhu (IHVN Institute of Human Virology); Collins John, Nathan Shehu (Jos University Teaching Hospital);

**Pakistan**: Assad Hafeez (Health Services Academy Ministry of Health); Abdul Wali Khan, Manohar Lal (Federal General Polyclinic Services Hospital); Prof Dr. Jai Krishin (Pakistan Institute of Medical Sciences); Ramesh Kumar (Health Services Academy); Zafar Fatmi (Aga Khan University); Rashid Qadeer, Malaika Jawaid Siddiqui, Erfa Tahir (Dr. Ruth Pfau Civil Hospital); Ashraf Jahangeer Al’Saani, Ramsha Zafar, Asma Burney (Dow University Hospital); Ayesha Iqbal (Services Institute of Medical Sciences Lahore); Zair Hassan (Lady Reading Hospital); Iftikhar Ali (Paraplegic Centre Hayatabad Peshawar); Muneeb Jan (Khyber teaching hospital, Peshawar); Mehreen Zakir (Sir Syed Hospital); Tehnia Nuzhat (Maternity and Child health center Abdullah Gabol Goth);

**Palestine**: Omar Riad Alnajjar, Ibrahim Tawfeeq Abdulhadimaqadma, Nour Abdallah abo Shawish, Minyar Ismael Wali, Hitham ibrahem toman, Aya Yassin, Ali wael alhabash, Ahmed Bassam Abu Thaher, Ruba Shaqoura, Mohammed abo abdo, Marah Emad Abu Selmiya, Abd AlRahman Alashqer, Remah Tayseer Jneed, Ahlam Shaheen, Ahmed Al-azzamy, Maryam Shaheen, Eman Jalhum, Mahmoud Ahmad Abo Ouda, Shadi Nahed Aljerjawi, Alaa Naser Alsharef, Ahmed Emad Habboub, Ameer Helles, Motaz Zakaria‎‏ Hammad, Osama Sweid (Islamic University of Gaza); Asil Nahed Aljerjawi, Salsabeel Hani Alhaw(Alazhar University of Gaza); Faris Hammad, Ihab N. Tahboub, Amjad Samara, Ala'a Samir Mohammad Alfuqaha, Moutaz W. Sweileh (An-Najah National University); Hasan Arafat (Istishari Arab Hospital);

**Paraguay**: Carlos Miguel Rios-González, Pablo Martinez Acosta (National University of Caaguazu);

**Philippines**: Paul Adrian Vidanes Pinlac, Angelica Anne Eligado Latorre, Kim Carmela Dee Co, Sharon Yvette Angelina Manalo Villanueva, Maria Margarita Maiquez Lota, Evalyn Alarkon Roxas (University of the Philippines Manila); Harold Robin Facing Laroa (Ospital ng Maynila Medical Center); Manila T. Villalon (Laguna Medical Center); Jehiel L. Fabon (Novaliches District Hospital); Denim E. Maghanoy (The Barrio Program Rural Health Physician); Nerissa G. Sabarre (Pasig City General Hospital);

**Russia**: Valeriya Mikhailova (First Moscow State Medical University); Mahir Gachabayov, Abakar Abdullaev (Vladimir City Emergency Hospital);

**Serbia**: Srdjan Stefanovic, Slobodan Jankovic, Milos Milosavljevic (University of Kragujevac); Danica Radomirovic, Milica Milosavljevic (General (Regional) Hospital of Kosovska Mitrovica);

**Somalia**: Abdulfatah Hassan Dhaqane, Hassan Yusuf Roble, Abdifatah Abdullahi Jalei, (Somali Sudanese Specialized Hospital), Shyam Prakash Dumre (Nagasaki University);

**South Africa**: Indiran Govender, Brett van Coppenhagen, Olga Maphasha (University of Pretoria);

**Spain**: Francesc Figueras Retuerta, Maria Dolores Gomez Roig (Institut Clínic de Ginecologia, Obstetricia i Neonatologia, Universitat de Barcelona); Rafael Sierra (Puerta del Mar University Hospital); Eva del Amo (Vall d'Hebron institut de Reçerca. Barcelona); Magda Campins (Vall d'Hebron institut de Reçerca. Barcelona);

**Sri** **Lanka**: H.S.K.Seneviratne, Ayodhya Priyadharshi Senanayake (Sirimavo Bandaranaike Specialized Children Hospital (SBSCH)), Chamara Dalugama (Teaching (General) Hospital-Peradeniya); DRRHB Dissanayake, MRM Rizky Aazath (District General Hospital-Nuwara Eliya); WMNP Buddhadasa, B.M.L.S.Basnayake (National Hospital-Kandy); WALP Weerasekara (District General Hospital-Trincomalee); KGCYSB Weerakoon (District Base Hospital-Teldeniya); Saranga Sumathipala, K.T.Rajith Jayasanka , Thilini Premadasa (Teaching Hospital Anuradhapura); Vaithehi Francis (Eastern University of Sri Lanka);

**Sudan**: Goutoof Abdelkhalig Elemam Yousif, Hadeel Hamza Mohammed Abaker, Mohamed Daffalla Awadalla Gismalla, Adam Mohamed Ibrahim Adam, Makarim Awad Moaz Omer, Rania Ahmed Elsiddig Hassan, Alaa Abaas Elsharief Mohammed Omer, Reham Abdelgader Alnaeem Babiker, Alkhansa Hamid Mohamed Alkhider, Elamin Abuobaida Elamin Mohamed, Maram Elgaylani Abdalla Eltayeb, Iman Hassan Mirgani Mohammed, Asma Mutasim Elrasheed Ghalib, Lamia MohyEldin Merghani Alkhder, Durar Amin Abdelbagi Musa, Mohammed Mutasim Ahmed Abudawa, Ola Mutasem Mohammed Dafallah, Omnia Ahmed Ben Ouf Mohamed (University of Gezira); Leena Hijazi Osman Ahmed (University of Kassala); Ibrahim Iyad Almharat, Khadiga Isameldin Elnour, Roa Abdel-Hafez Musa, Yousif Sultan Abdelrahim (University of Bakhtalruda); Samah Mohammed Ibrahim Zaroog, Fatima Hassan Mohammed Fadelalmawla (West Kordufan University); Namareg Mohamed Edris Khiri, Tagwaa Elser Mohammed Ahmed Babiker (University of Khartoum); Mohannad Shaban, Radi Tofaha Alhusseini, Mohammed Alfatih, Malik Ahmed Malik Ahmed, Mohamed Hamid Abdelsalam Mohamed, Mariam Mohamed Ali, Mohamed Faroug Ali Yassin, Jumaa Mohammed Abdallah Hamed, Ali Ahmed Rabih Idriss, Mohajer Ibrahim Hassan Ismaeil, Hussain Mohamed Ahmed Khalifa, Omer Saeed Mohamed Saeed, Ahmed Eltag Elnagi Adam, Mohamed Hajhamad Abdoalazez Mohamed Elamin, Yaseen Muhammed Abdelhafeez Hamid, Waad Elzamali Elmonower, Mohamed Abdulkarim Mohamed Abdulrahim, Baha Aldeen Abdalaziz Alshareif, Tebyan Elbagir Elshiekh Mohamed, Hajer Hatim Hassan Ahmed, Mohammed Atif Abbas Mohammed Ahmed (Alzaiem Alazhari University, Khartoum); Asrar Abobkr Ishag Dawood, Rashed Yagoub Adam Abdalla, Saddam Adam Alnour Mohammed, Mohammed Ibrahim Omer Abdalla (University of Bahri); Malaz Salah Osman Gurashi (University of Science and Technology); Rawan Raad Hassan Elrufai, Rawan Hassan Adm Idrys (Sudan University of Science and Technology); Alaa Eldirdiri Elgaili Eldirdiri (Ibn Sina University); Albokhary Salih Adam Abkar (Nahda College); Wishah Mohammednour Ahmed, Waad Eltayeb Ageib Elsheikh, Malaz NasrEldin Elaagib Ahmed, Elrasheed Ghalib Abdalrhman (Omdurman Islamic University); Tarig Abdelwahab Abdelrahim Elnour (Clinic of Khartoum Refinery Company);

**Syria**: Mosa Shibani, Abdallah Altorkmani, Ahmad Rmman, Bisher Sawaf, Mhd Amin Alzabibi, Hlma Ismail, Humam Armashi, Mohamad Ziab Chebat (Syrian Private University); Ahmad Alkhaledi, Danny Hadidi, Hind Kazem, Hiba Mardini, Sara Naoura, Omar Alneser, MHD Bahaa Aldin Alhaffar, Aram Abbas, Ibrahim Alorfhli, Ibrahim Shammas, Osama Al Habbal (Damascus University);

**Taiwan**: Nai-Ying Ko, Nan-Yao Lee (National Cheng Kung University Hospital);

**Tanzania**: Jaffu Othniel Chilongola, Florida Muro (Kilimanjaro Christian Medical Center);

**Thailand**: Krongkarn Sutham, Poramed Winichakoon, Chidchanok Ruengorn, Ratanaporn Awiphan (Chiang Mai University); Kittima Bangpattanasiri, Piamlarp Sangsayunh, Sakkarin Kungsukool, Anek Kanoksilp (Central Chest Institute of Thailand); Watsamon Jantarabenjakul, Opass Putcharoen, Leilani Paitoonpong, Gompol Suwanpimonkul (King Chulalongkorn Memorial Hospital); Thanyawee Puthanakit (Chulalongkorn University); Suparat Kanjanavanit, Rathakarn Kawila (Nakornping Hospital); Woravut Kowatcharakul (Sansai Hospital); Sirisak Nanta (Maesai Hospital);

**Trinidad & Tobago**: Rajeev Peeyush Nagassar, Darren Kendall Dookeeram, Keston Daniel, Saskia Ramkissoon-Bain (Eastern Regional Health Authority (ERHA)); Shyam Prakash Dumre (Nagasaki University);

**Tunisia**: Hamida Maghraoui (Rabta University Hospital); Amal Jebali, Salma Ben Saber, Wajdi Kacem, Nacir Dhouibi, Nouha Dissem, Ahmed Nefzi, Farah Bahri, Majdi Ben Romdhane, Faten Tlili (University of Tunis El Manar); Riadh Ben Rhaiem, Ines Trabelsi (University of Monastir); Hanen Ben Ammar, Emira Khelifa (Tunis El Manar University Razi Hospital); Zied Moatemri (Principal Military Hospital of Instruction of Tunis); Kawthar Dhib, Omar Mastouri, Chams Zarrad (University of Sousse); Ahlem Aissa (Enfidha Hospitals); Zeineb Teyeb, Abir Malouch (Hospital of Intern Security Forces); Mouna Gara, Asma Ladib, Lotfi Grati (El Omrane Hospital); Omar Ammous, Zahra Charmi, Mohamed Ksentini (Faculty of Medicine, University of Sfax);

**United Arab Emirates**: Amina AI-Marzouqi (University of Sharjah-University Hospital);

**Ukraine**: Olha Tkachuk (Bogomolets National Medical University); Iryna Kudlatska-Tyshko, Ali Dzhemiliev (Shupyk National Medical Academy of Postgraduate Education);

**United States of America**: George L. Anesi (Hospital of the University of Pennsylvania); Robyn Scatena, Pavan V. Ganapathiraju (Norwalk Hospital & Danbury Hospital); Kelley M. Anderson (MedStar, Washington Hospital Center);

**Vietnam**: Le Ngoc Phu, Pham Thi Minh Chau, Tran Hoang Thanh Hang (Danang Hospital); Dinh Thanh Nhan, Nguyen Thi Phuong Diem (Family Hospital); Le Van Cuong, Le Van Sy, Do Xuan Tien (Thanh Hoa General Hospital); Nguyen Thi Minh Trang, Nguyen Thi Mai, Duong Thanh Trang Dai (Quang Nam Hospital for Women and Children); Ho Van Han, To Le Na, Dang Phuong Thinh (Nhan Dan Gia Dinh Hospital); Vo Nguyen Trung, Ha Manh Tuan (University Medical Center Campus No.2); Nguyen Minh Tuan, Du Tuan Quy, Truong Huu Khanh, Nguyen Thanh Hung, Ngo Ngoc Quang Minh (Children's Hospital 1); Trinh Huu Tung, Pham Thai Son, Nguyen Xuan Thuy Quynh, Do Chau Viet, Nguyen Minh Ngoc (Children's Hospital 2); Nguyen Thi Hien, Tran Quoc Hung, Nguyen Thi Hoai Phuong (Hue City Hospital); Nguyen Ngoc Linh, Nguyen Quoc Thang, Nguyen Tien Manh (My Duc Psychiatric Hospital); Nguyen Van Tien (Quynh Phu General Hospital); Ta Van Tram, Do Quang Thanh, Le Quang Tin (Tien Giang Center General Hospital); Mai Ngoc Luu, Lam Hoang Cat Tien, Le Thi Thu Thao, Nguyen Thanh Van (Nguyen Tri Phuong Hospital); Nguyen Van Dang, Truong Xuan Hung (199 Hospital); Vu Nam, Tran Thai Ha, Vu Thi Thu Trang (National hospital of traditional medicine); Le Thi Hoai Anh, Le Thi Thuy, Le Van Truong (Traditional Medicine Hospital of Ministry of Public Security, Vietnam); Tran-Thuy Nguyen, Nguyen Cong Huu, Pham Thi Thoa (E Hospital); Le Duc Tho, Pham Le Xuan Huy, Nguyen Van Thoai (Military Hospital 211); Vo Thi Thuy, Le Quang Duan, Doan Van Hoai Nam (Nghe An Northwest Regional Hospital); Nguyen Tri Thuc, Le Quoc Hung, Phung Manh Thang, Lam Viet Trung, Dang Vu Thong (Cho Ray Hospital); Le Thuong Vu (University of Medicine and Pharmacy at Ho Chi Minh city); Nguyen Duc Truong, Vu Truong Son, Tran Xuan Tiem (FV Hospital); Phan Thi Hang, Hoang Thi Diem Tuyet (Hung Vuong Hospital); Hoai-Nam Thai (University Medical Center Ho Chi Minh City), Dieu-Thuong Thi Trinh, Van-Dan Nguyen, Minh-Man Pham Bui (University of Medicine and Pharmacy at Ho Chi Minh City); Nguyen Thanh Phuoc, Dao Anh Dung (Cao Van Chi Hospital, Tay Ninh); Nguyen Tran Nam, Nguyen Trong Nhan, Vuong Kien Thanh, Lam Tuyet Trinh (City Children's Hospital of Ho Chi Minh City);

**Zambia**: Duncan Chanda (University Teaching Hospital, Lusaka); Bright Nsokolo (Levy Mwanawasa General Hospital); Gershom Chongwe (Ndola Teaching Hospital); Katongo Mutengo (Livingstone Central Hospital).

**Protocol and questionnaire translators:**

**Albanian**: Irida Dajti, Jola Kërpaçi, Enxhi Vrapi, Jehona Neziraj, Eldona Hajdinaj, Klea Beqiraj, Amina Cani, Anila Sinani, Lorenc Bellani;

**Arabic**: Mona Hanafy Mahmoud, Kirellos Said Abbas, Fatima A. Monib, Dina Emad, Mina Ragy, Mirna Fawzy, Moosa Hani Shibani, Hosam Waleed Shaikhkhalil;

**Bengali**: Sajibur Rahman, Orindom Shing Pulock, Farhana Nusrat, Abdur Rafi, Ima Islam, Nahida Hannan Nishat, Muhammad Rashidul Hashan, Bishwanath Acharya, Shyam Prakash Dumre;

**Chinese**: Lim Yi Liang, Brenda Phang Joo Yee, Tan Geok Eng, Juwie Chuah, Phoong Yu Lian, Yap Siang Jee, Li Chuin Chong, Ahmad Taysir Atieh Qarawi, Ng Sze Jia, Nguyen Hai Nam;

**French**: Ahmad Taysir Atieh Qarawi, Nacir Dhouibi, Wiem Belhadj, Ahmed Nefzi, Fatin Tlili, Nesrine Ben Hadj Dahman, Nouha Dissem, Salma Ben Saber, Oumaima Outani, Zaineb Ben Slimane;

**Hindi**: Akash Sharma, Akshay Raut, Suhrud Panchawagh, Adnan Mansuri;

**Indonesian**: Ichan Pan, Harapan Harapan, Ally Molly;

**Italian**: Simone Piva, Daniela Acquadro Maran, Maria Michela Gianino, Fabio Porru, Riccardo Papalia;

**Japanese**: Koji Aoki, Akiko Saito, Yoshimi Nitta, Atsuko Imoto;

**Kurdish**: Trifa Abdalla Mahmood, Heshu Rahman, Darya Saeed, Kawa Faray, Dana Taib, Jeza Muhamad Abdul Aziz, Hemn Othman;

**Nepali**: Kamal Ranabhat, Renu Bhandari, Dhiraj Acharya, Bishwanath Acharya, Kishor Pandey, Shyam Prakash Dumre;

**Persian**: Fatemeh Oskouie, Fereydoon Khayeri, Naiemeh Seyedfatemi, Nahla Farhani, Sedighe Karimzadeh, Mona Mahmoud;

**Portuguese**: Filipa Ribeiro Lucas, Maria Margarida Ribeiro, Bernardo Soares;

**Russian**: Mariia Pavlenko, Dmytro Pavlenko, Roman Pavlenko;

**Spanish**: Julio Aguilar Morales, Antonio M Quispe, Briseida Jiménez Velázquez, María Fernanda Paz y Miño, Diana Lip Sosa, Riagam Jafet Martinez Portilla;

**Thai**: Nut Koonrungsesomboon, Watsamon Jantarabenjakul, Bishwanath Acharya, Shyam Prakash Dumre;

**Urdu**: Usman Ghani, Muhammad Saeed, Somia Iqtadar;

**Vietnamese**: Vuong Thanh Huan, Truc Phan, Mai Ngoc Luu, Vu Quoc Dat, Hoang Thi Nam Giang, Vuong Ngoc Thao Uyen, Hoang-Minh NGUYEN, Tran Nu Thuy Dung, Nguyen Phuc Nguyet Anh, Pham Thai Son, Minh-Man Pham Bui.

**Data Validators: (checking & converting data team)**

**Afghanistan:** Tran Nu Thuy Dung;

**Albania:** Irida Dajti, Jola Kërpaçi, Enxhi Vrapi, Tran Nu Thuy Dung;

**Algeria:** Tran Nu Thuy Dung, Trang Huynh;

**Bangladesh:** Mennatullah Mohamed Eltaras;

**Cameroon:** Hoang-Minh NGUYEN;

**Chile:** Le Van Truong, Nguyen Thi Linh Hue;

**China:** Yap Siang Jee; Vy Thi Nhat Huynh, Hoang-Minh NGUYEN;

**Ecuador:** Raigam Jafet Martinez-Portilla, Tran Nu Thuy Dung;

**Egypt:** Mohammed Soliman, Mennatullah Mohamed Eltaras, Shamael Thabit Mohammed Alhady, Ezz eldeen Atef Derballa, Asmaa Khaled Mostafa, Nada khaled Abdelsattar, Abdullah Ahmed Ahmed, Taha Abdelsalam Ashraf Taha, Mahmoud Ahmed Farag, Hanaa Abdelmonem Hussien, Ahmed Taher Masoud, Mohamed Abdelmonem, Manar Hamdy Mohammed, Hoang-Minh NGUYEN, Trang Huynh;

**Ethiopia:** Hai-Yen Tran; Mohammed Soliman, Shamael Thabit Mohammed Alhady, Mennatullah Mohamed Eltaras;

**France:** Tran Nu Thuy Dung;

**Greece:** Le Van Truong, Vu Thi Thu Trang;

**Guatemala:** Raigam Jafet Martinez-Portilla, Tran Nu Thuy Dung;

**India:** Akash Sharma, Nguyen Thi Linh Hue, Yap Siang Jee, Mallak Alomoush, Mona Hanafy Mahmoud;

**Indonesia:** Kadek Agus Surya Dila (Pratama Giri Emas Hospital);

**Iran:** Tran Nu Thuy Dung;

**Iraq:** Jeza Muhamad Abdul Aziz, Tran Nu Thuy Dung;

**Japan:** Vu Thi Thu Trang, Atsuko Imoto, Le Van Truong, Koji Aoki, Akiko Saito;

**Jordan:** Mohammed Soliman, Mallak Alomoush, Hoang-Minh NGUYEN;

**Lebanon:** Trang Huynh;

**Malaysia:** Nguyen Anh Thi, Vy Thi Nhat Huynh, Dang Thuy Ha Phuong, Mohammed Soliman, Tran Thuy Huong Quynh, Vuong Ngoc Thao Uyen;

**Mexico:** Raigam Jafet Martinez-Portilla, Nguyen Thi Linh Hue, Le Van Truong;

**Morocco:** Hoang-Minh NGUYEN, Trang Huynh;

**Nepal:** Tran Nu Thuy Dung;

**New Zealand:** Trang Huynh;

**Nigeria:** Vu Thi Thu Trang, Le Van Truong;

**Pakistan:** Vu Thi Thu Trang, Le Van Truong;

**Palestine:** Hosam Waleed Shaikhkhalil;

**Paraguay:** Le Van Truong, Vu Thi Thu Trang;

**Philippines:** Luu Ngoc Mai, Vuong Ngoc Thao Uyen, Vy Thi Nhat Huynh, Tran Thuy Huong Quynh, Nguyen Anh Thi;

**Russia:** Dmytro Pavlenko, Nguyen Thi Linh Hue;

**Serbia:** Trang Huynh;

**South Africa:** Vu Thi Thu Trang, Le Van Truong;

**Spain:** Raigam Jafet Martinez-Portilla, Hai-Yen Tran;

**Sri Lanka:** Hai-Yen Tran;

**Sudan:** Mona Hanafy Mahmoud, Jeza Muhamad Abdul Aziz, Mohammed Soliman, Mennatullah Mohamed Eltaras, Pham Dinh Long Hung, Vuong Ngoc Thao Uyen;

**Syria:** Basel Kouz, Ahmad Alkhaledi, MHD Bahaa Aldin Alhaffar, Hoang-Minh NGUYEN, Trang Huynh;

**Taiwan:** Vuong Ngoc Thao Uyen;

**Tanzania:** Le Van Truong, Vu Thi Thu Trang;

**Thailand:** Luu Ngoc Mai, Pham Dinh Long Hung, Dang Thuy Ha Phuong;

**Tunisia:** Tran Nu Thuy Dung, Hoang-Minh NGUYEN, Trang Huynh;

**United Arab Emirates:** Le Van Truong, Vu Thi Thu Trang;

**Ukraine:** Dmytro Pavlenko, Nguyen Thi Linh Hue;

**United States of America:** Le Van Truong, Vu Thi Thu Trang;

**Vietnam:** Tran Thuy Huong Quynh; Yen-Xuan Thi Nguyen; Luong Thi Trang; Pham Dinh Long Hung;

**Zambia:** Vu Thi Thu Trang, Le Van Truong;
